# Assessing the prognostic utility of clinical and radiomic features for COVID-19 patients admitted to ICU: challenges and lessons learned

**DOI:** 10.1162/99608f92.9d86a749

**Published:** 2024-01-31

**Authors:** Yuming Sun, Stephen Salerno, Ziyang Pan, Eileen Yang, Chinakorn Sujimongkol, Jiyeon Song, Xinan Wang, Peisong Han, Donglin Zeng, Jian Kang, David C. Christiani, Yi Li

**Affiliations:** † Biostatistics, University of Michigan, Ann Arbor, MI; ‡ Environmental Health, Harvard T. H. Chan School of Public Health, Boston, MA

**Keywords:** COVID-19, machine learning, survival prediction, X-ray imaging

## Abstract

Severe cases of COVID-19 often necessitate escalation to the Intensive Care Unit (ICU), where patients may face grave outcomes, including mortality. Chest X-rays play a crucial role in the diagnostic process for evaluating COVID-19 patients. Our collaborative efforts with Michigan Medicine in monitoring patient outcomes within the ICU have motivated us to investigate the potential advantages of incorporating clinical information and chest X-ray images for predicting patient outcomes. We propose an analytical workflow to address challenges such as the absence of standardized approaches for image pre-processing and data utilization. We then propose an ensemble learning approach designed to maximize the information derived from multiple prediction algorithms. This entails optimizing the weights within the ensemble and considering the common variability present in individual risk scores. Our simulations demonstrate the superior performance of this weighted ensemble averaging approach across various scenarios. We apply this refined ensemble methodology to analyze post-ICU COVID-19 mortality, an occurrence observed in 21% of COVID-19 patients admitted to the ICU at Michigan Medicine. Our findings reveal substantial performance improvement when incorporating imaging data compared to models trained solely on clinical risk factors. Furthermore, the addition of radiomic features yields even larger enhancements, particularly among older and more medically compromised patients. These results may carry implications for enhancing patient outcomes in similar clinical contexts.

## Media Summary

Severe cases of COVID-19 often require intensive care unit (ICU) escalation, where patients may still experience serious disease courses and outcomes, including mortality. Reliable predictors of disease severity would be invaluable for clinicians to evaluate these patients and improve treatment and management. However, limited work has been done to assess clinical outcomes among COVID-19 ICU patients. Chest X-rays are an essential part of diagnostic practice in evaluating patients with respiratory infections, including COVID-19. Our team has partnered with the University of Michigan Health System (or Michigan Medicine) throughout the pandemic to monitor the ICU outcomes of COVID-19 patients using DataDirect, a GPU-based analytics platform launched by Michigan Medicine. Our experience has enabled us to explore the potential benefits of using both clinical information and chest X-ray images to predict COVID-19 outcomes among these patients with severe infection. Combining these data sources via DataDirect, we aim to develop more accurate prediction models that can aid in clinical decision-making and ultimately improve ICU outcomes. We propose an analytical workflow to address challenges such as the absence of standardized approaches for image pre-processing and data utilization. We then propose an ensemble learning approach designed to maximize the information derived from multiple prediction algorithms. Our results show that using this ensemble method to integrate clinical and imaging data sources can lead to more accurate predictions of ICU mortality, especially among older and sicker patients.

## 1. Covid-19 Ehr Data, Analytical Workflow of Extracting Radiomic Features, and Ensemble Prediction

COVID-19 has undeniably reshaped the realm of critical care, shedding light on challenges pertaining to the clinical comprehension of this ailment and the statistical intricacies intertwined with data collection, processing, and analysis. Throughout the pandemic, our team collaborated with the University of Michigan Health System (Michigan Medicine) to investigate the risk factors associated with disease severity and patient outcomes. Our earlier work studied whether risk factors for COVID-19 that were identified during the initial wave persisted in the first year of the pandemic and across outcomes of varying severity (Salerno, Sun, et al., 2021). We found differences in the frequency of healthcare utilization and more severe COVID-19 outcomes such as hospitalization, readmission, and mortality, as well as differing risk factors for these outcomes, particularly when comparing younger, non-Black patients to older, male, and Black patients, as well as when comparing patients of differing comorbidity burden. As severe cases of COVID-19 often necessitate escalation to the intensive care unit (ICU), where patients may continue to face critical disease courses and outcomes, we anticipate that expanding our prior research to an ICU context could offer valuable insights to healthcare providers in emergency departments and critical care settings regarding treatment priorities. A pivotal advancement in this work is to harness a new analytical workflow and machine learning algorithm, enabling us to make full use of the extensive data available within Michigan Medicine’s electronic health record (EHR) as well as the patient images facilitated by DataDirect within an ICU setting.

### 1.1. DataDirect and the Democratization of Michigan’s EHR.

DataDirect is a GPU-based analytics platform launched by Michigan Medicine to allow researchers to collaborate on datadriven analyses for disease prevention and treatment through the shared EHR data of the entire hospital system (DataDirect, 2023). This rich database provides health and geolocation data of more than 4 million Michigan Medicine patients, as well as an imaging data repository of more than 750,000 chest X-rays for 100,000+ patients, genetic testing results, and patient-reported survey data. Moreover, chest X-rays are an essential part of diagnostic practice in evaluating patients with respiratory infections, such as COVID-19. As part of the Precision Health Initiative, Michigan Medicine has collected X-ray images from inpatient settings, including those patients with COVID-19. As portable chest X-rays are efficient in triaging emergent cases, their use has raised the question of whether imaging carries additional prognostic utility for survival among patients with COVID-19. With access to the EHR and X-ray data from these sources via DataDirect, we have been in a unique position to develop new methodologies for identifying patient characteristics, clinical factors, and radiomic features linked to COVID-19 status, disease severity, and survival outcomes, and to evaluate the efficacy of ensemble learning methods for COVID-19 patient risk stratification and prognostication. Our prior work focused on machine learning techniques to assess the prognostic utility of radiomic features for in-hospital COVID-19 mortality (Sun et al., 2022). Our study found incremental improvements in prognostic ability utilizing texture features derived from X-rays, and we concluded that chest X-rays, in conjunction with clinical information, may be predictive of survival outcomes particularly among older patients or those with higher comorbidity burden.

### 1.2. Proposed Reproducible Analytic Workflow.

This experience has enabled us to scrutinize the benefits of using both radiomic features and clinical information when building predictive models. In this work, we focus on formalizing the task of prediction in settings where readily available radiomic data, such as images taken via portable chest X-ray, may supplement or even replace clinical information which would be taken during an extended history and physical examination, that may be unavailable in emergent or critical care settings (Ramani et al., 2021). Owing to the wealth of data available through DataDirect, we have extracted and created a set of demographic, socioeconomic, and clinical risk factors which have previously been identified as being related to COVID-19 in the literature. In response to the unique challenges associated with chest X-ray data for COVID-19, that is, no available image segmentation information, we propose a principled pipeline for feature extraction with the X-ray data, where we select relevant imaging features based on patient survival information.

### 1.3. Weighted Ensemble Averaging.

To gain a deeper insight into the potential impact of employing diverse predictive modeling strategies on our research outcomes, we conducted a comprehensive comparison of four commonly-utilized prediction algorithms through a series of simulations. These simulations were designed to shed light on the performance variations among these algorithms and their potential applications in our study. Initially, we created an ensemble learner by averaging the risk scores generated by each individual learner, which, as a proof of concept, allowed us to assess whether a “collective wisdom” approach could outperform individual predictors.

Subsequently, we took a more rigorous approach by proposing an ensemble learner that harnessed the maximal information from these individual strategies. This involved optimizing the ensembling weights and considering the shared variability of the individual risk scores. Our simulations demonstrated the superior performance of this weighted ensemble averaging approach across a spectrum of scenarios. We then applied this refined ensemble methodology to analyze post-ICU COVID-19 mortality, which we observed in 21% of patients with COVID-19 in acute care settings at Michigan Medicine. By leveraging this ensemble method, we were able to construct predictions using the DataDirect platform while fostering collaboration with Michigan Medicine for this pivotal project.

While the integration of data from various sources has been explored extensively in precision oncology and other fields, we present our work in a pulmonary critical care setting, which may also provide a compelling use case for such integration. Our study showcases the application of machine learning in this critical healthcare setting and delves into the construction of a dependable ensemble risk score. These insights may be valuable for advancing our understanding of predictive modeling and hold significant implications for improving patient outcomes in similar clinical contexts.

## 2. Motivating Data

Coronavirus disease 2019 (COVID-19) is a respiratory illness that presents with a wide range of symptoms and clinical manifestations (Hoogenboom et al., 2021; Karagiannidis et al., 2021). Though the impact and severity of the COVID-19 pandemic have varied in the past three years, a significant number of COVID-19 patients experience rapid progression of respiratory compromise and other complications, leading to mechanical ventilation and intensive care unit (ICU) admissions (Chang et al., 2021; Hosey & Needham, 2020). These patients experience serious disease courses and outcomes, including mortality, which ranged from 25.7% to 28.3% (McCue et al., 2021; Quah et al., 2020), with some reports citing rates as high as 100% (Michelen et al., 2021). Reliable predictors of disease severity would be invaluable for assessing COVID-19 ICU patients, and enhancing treatment and management. However, limited research has been done to evaluate clinical outcomes among these ICU patients. Moreover, since the start of the pandemic, several dominant variants have arisen, leading to modifications in symptom management and therapeutic protocols (Dutta, 2022; L. Lin et al., 2022). There is limited research on the variation in predictors among severe COVID-19 patients across different variants (Ayala et al., 2021; El-Shabasy et al., 2022). The data outlined below pertain to our comprehension of the mortality risk factors for patients with COVID-19 following their admission to an ICU.

### 2.1. Study Population and Outcome.

The eligibility criteria for participants in this study encompass patients who meet all of the following conditions: (1) confirmed positive for COVID-19 or transferred with a confirmed positive diagnosis, (2) were hospitalized in a Michigan Medicine ICU between March 10, 2020, and January 26, 2022, and (3) possessed at least one COVID-related chest X-ray image on record (Jiao et al., 2021). A total of 2,289 patients meeting these inclusion criteria were included in the study; see Figure 4 for the derivation of our study cohort. The primary outcome is post-ICU mortality, defined as the time from first ICU admission due to COVID-19 until death, which could be censored by the end of the follow-up window. Our methods and findings are limited to this specific population, although the general methodological framework may be adaptable to other comparable settings.

### 2.2. Potential Risk Factors.

We collected temporal information on COVID-19 diagnosis, COVID-19 related ICU-escalation, and death (where applicable) from the DataDirect database. In addition, we collected EHR-derived risk factors, including patient demographics, socio-economic status, comorbidity conditions, vaccination records, and physiologic measurements. Patient demographics included age, sex, self-reported race and ethnicity, smoking status, alcohol use, drug use, and COVID-19 vaccination status. We defined vaccination status based on recorded vaccine doses and types, i.e., 0 = ‘Not Vaccinated’ (no doses before first ICU escalation), 1 = ‘Partially Vaccinated’ (one dose of Moderna or Pfizer), or 2 = ‘Fully Vaccinated’ (two doses of Moderna or Pfizer, or one dose of Janssen).

We defined twenty-nine prevalent comorbidity conditions based on whether the patient had any associated ICD-10 codes on admission. We further obtained physiologic measurements within 24 hours of ICU escalation, including body mass index, oxygen saturation, body temperature, respiratory rate, diastolic and systolic blood pressure, heart rate, and need for respiratory support such as mechanical ventilation. We used patient residences to define neighborhood socioeconomic status at the US census tract-level. We defined four composite measures based on the average proportion of adults within a given census tract meeting certain criteria for (1) affluence, (2) disadvantage, (3) ethnic immigrant concentration, and (4) education level, categorized by quartiles (Salerno, Sun, et al., 2021; Salerno, Zhao, et al., 2021; Sun et al., 2022). See Appendix A for details. We excluded potential risk factors with sizable missing data rates >30%, such as demographic and social history data (e.g., marital status) and certain patient care measurements (e.g., invasive vital sign measurements); otherwise, to fill in missing values, we used mean or mode imputation for computational convenience. Among those predictors included in our subsequent modeling, missingness rates varied from 4.19% (BMI) to 19.05% (body temperature). A full summary of these missingness rates among all potential risk factors can be found in Appendix B, Table B1.

**Figure 1.**
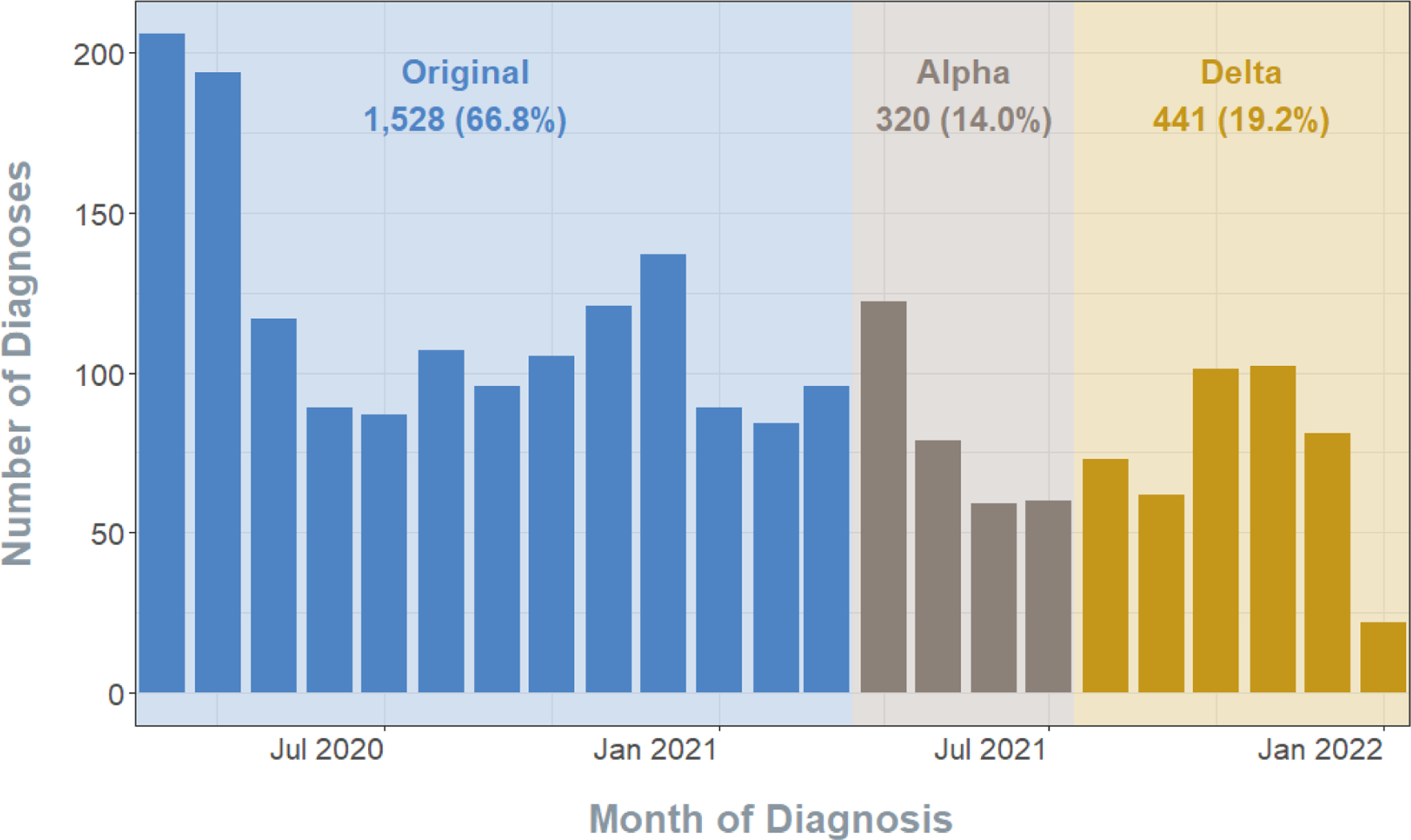
Distribution of COVID-19 diagnoses by the time period for the 2,289 patients admitted to a Michigan Medicine ICU.

To address ongoing concerns about new mutations and the potential utility of our proposed approach in the future, we included the dominant variant period of the virus at the time of infection as another possible predictor (refer to Figure 1). We defined the *dominant variant period* based on a patient’s date of COVID-19 diagnosis, with the following categories: Original (March 2020 - March 2021), Alpha (April 2021 - July 2021), and Delta (August 2021 - January 2022). This variable was intended to serve as a proxy for the impact of the particular wave of the pandemic, which may contain variations in the virus, disease severity, and provided therapeutic interventions and care standards. As explained later, we conducted sensitivity analyses on our proposed approach with respect to this variable to determine whether its prognostic value may change.

All patients in our study had at least one COVID-related chest X-ray image, which was taken in either the anterior-posterior or left-right axes, based on the anatomical coordinate system. In cases where patients had images taken from multiple orientations, we only considered those taken from the anterior-posterior or posterior-anterior positions, as these images had the same orientation and were the most prevalent. Our analysis used the images taken closest to the time of ICU admission.

## 3. Proposed Reproducible Analytic Workflow

**Figure 2.**
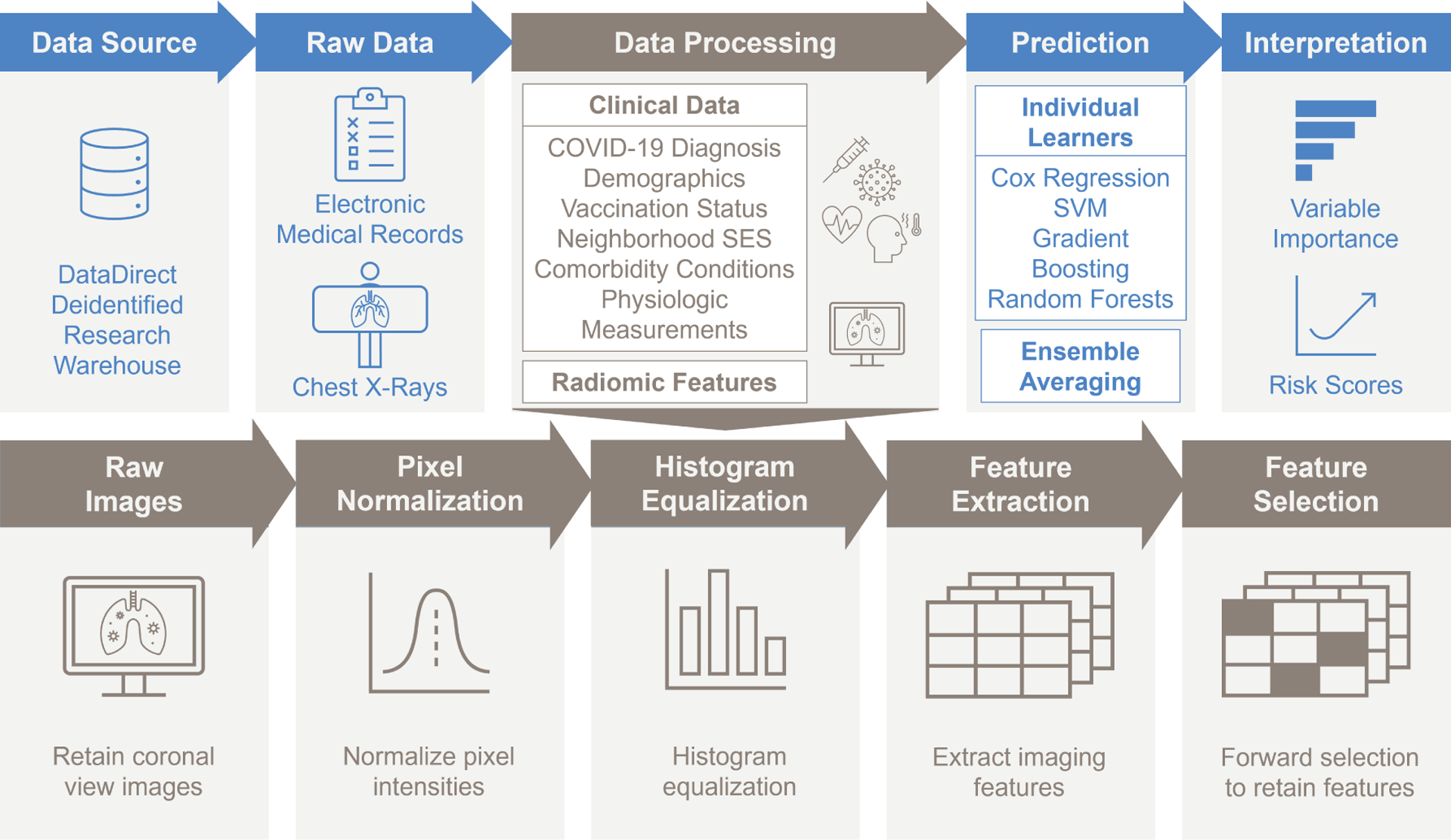
Flowchart of data processing and analytic workflow

The clinical and imaging data were obtained from the Precision Health DataDirect Deidentified Research Warehouse. The clinical features were aggregated either at the patient-encounter or patient-order level. The raw Digital Imaging and Communications in Medicine (DICOM) image files and their corresponding image headers, which were identified using accession numbers, were linked to the patient-encounter data. The data was pre-processed as described in this study before use in our predictive models; see Figure 2.

### 3.1. Image Pre-Processing.

To address the lack of available image segmentation information for COVID-19 chest X-rays, as well as the high variability in the characteristic reticular “ground glass” opacifications, we propose a principled pipeline for feature extraction with the X-ray data, where we select relevant imaging features based on patient survival information. We pre-processed each image according to the pipeline in Figure 2. After selecting the appropriate raw image files, we normalized the pixel intensities of each image to a standard range of 0 (black) to 255 (white) units. This allows for the pixel information to be stored with less memory, facilitating more efficient computation. We then used histogram equalization to enhance the contrast of the images, by “spreading out” frequent pixel intensity values and the range of the image intensities (Jain, 1989).

As opposed to directly using the image pixel data in our predictive methods, we extracted texture features from the images. Texture features summarize the image characteristics, namely the spatial distribution of the pixel intensity levels (Galloway, 1975; Haralick et al., 1973). We extracted seven feature classes from each image: (1) first order, (2) shape, (3) gray level co-occurrence matrix, (4) gray level size zone matrix, (5) gray level run length matrix, (6) neighboring gray tone difference matrix, and (7) gray level dependence matrix (Chu et al., 1990; Thibault et al., 2013). In addition to the texture features extracted from the original, pre-processed images, we also extracted higher-order features from the images after applying six different filters: (1) wavelet, (2) Laplacian of Gaussian, (3) square, (4) square root, (5) logarithm, and (6) exponential. With seven classes of features extracted from the original and six transformed images, we obtained a total of 1,311 candidate image features using pyradiomics (van Griethuysen et al., 2017).

### 3.2. Feature Screening.

After image pre-processing, we obtained a texture feature matrix for each patient, from which we further selected target radiomic features that reflected patterns related to patient survival. The goal of this initial feature screening was to generate more interpretable and parsimonious prediction models. We first selected candidate features by fitting univariate Cox proportional hazards models on each feature (Therneau & Grambsch, 2000), retaining those that were statistically significant (p-value ≤ 0.05). To prevent information leakage, we did not perform feature screening and selection using all the data. Instead, in each experiment, we used the training data to do feature screening, feature selection, and modeling fitting, while the predictive performance of each method was calculated on the testing data. To further explore the impact of the clinical and demographic covariates on the selection of the radiomic features, and to assess any potential overlap of predictive information in these features with the clinical data, we performed a sensitivity analysis by adjusting for these variables during later model fitting and feature selection.

### 3.3. Example Patient Image Features.

**Figure 3.**
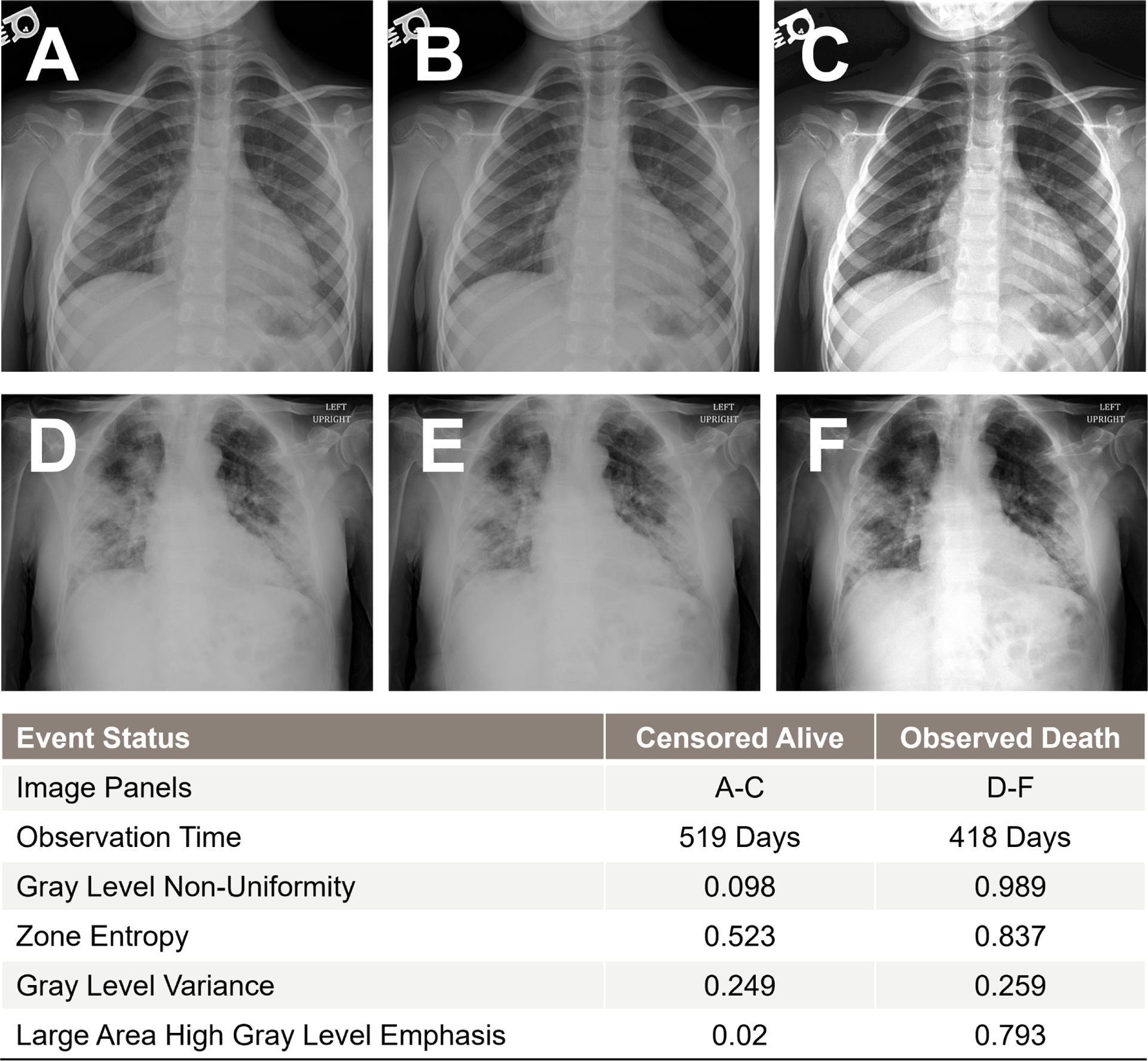
Raw images and pre-processed images along with extracted radiomic features for two example patients, one patient who was observed alive at the end of follow-up (Row 1, Panels A-C), and one patient who died during the follow-up period (Row 2, Panels D-F). Selected imaging features are given for comparison.

We exemplify the image pre-processing and feature extraction in two random patients selected from the study population – one who died during the follow-up period, and one who did not (i.e., censored; see Figure 3). The patient who died had higher values in the extracted texture features, namely the gray level non-uniformity (0.989 versus 0.098), zone entropy (0.837 versus 0.523), gray level variance (0.259 versus 0.249), and large area high gray level emphasis (0.793 versus 0.02). Higher values in this context correspond to greater heterogeneity in the texture patterns, indicative of the characteristic bilateral airspace opacities.

### 3.4. Computational Resources.

We conducted our data processing and analysis using Python (version 3.9.7), along with key libraries such as NumPy (version 1.24.2) and scikit-survival (version 0.19.0). Data pre-processing and model training were conducted in a high-performance computing (HPC) environment consisting of administrative nodes and standard Linux-based server hardware housed in a secure data center. These components were interconnected via both a high-speed Ethernet network (1 Gbps) and an InfiniBand network (40/100Gbps). A compliant parallel file system, meeting HIPAA regulations, was available for temporary data storage to support research. The project utilized six dedicated nodes, each equipped with eight RTX2080Ti GPUs, totaling 48 GPUs. On average, it took 303 seconds (with a range of 288 to 318 seconds) or approximately five minutes to extract texture features from a single raw X-ray image across 100 replications.

## 4. Statistical Analysis

### 4.1. Methods.

We first considered several commonly used algorithms to construct our risk prediction models, namely, the Cox proportional hazards model (Therneau & Grambsch, 2000), survival support vector machines (Van Belle et al., 2007; Y. Wang et al., 2016), random survival forests (Ishwaran et al., 2008), and survival gradient boosting (Hothorn et al., 2006). We constructed an ensemble learner by averaging the risk scores from each of these four individual learners (Sun et al., 2022; Viana et al., 2009; P. Wang et al., 2019). We then proposed a more efficient ensemble learner tailored for survival analysis. This new ensemble method maximizes the utilization of information from the individual approaches by fine-tuning the ensembling weights and incorporating considerations for the shared variability present in the individual risk scores.

#### 4.1.1. Notation.

With right censoring, we let T and C denote survival time and censoring time, respectively. We observe Y=min(T,C), and δ=I(T≤C), where I(⋅) is the indicator function. We further assume the observed data $\left\{\left(Y_{i},\delta_{i},X_{i}\right),i=1,\ldots ,n\right\}$ are i.i.d. copies of (Y,δ,X), where $X_{i}={\left(X_{i,1},\ldots ,X_{i,p}\right)}^{⊤}\in R^{p}$ denotes the p–dimensional risk factors for each patient.

#### 4.1.2. Cox Proportional Hazards Regression.

The Cox model (Cox, 1972) specifies that, at time t, the conditional hazard of post-ICU mortality for a patient with a set of p risk factors, $X_{i}$, is

$$\lambda \left(t|X_{i}\right)=\lambda_{0}(t)exp\left(X_{i}^{⊤}\beta \right),$$

where $\lambda_{0}(t)$ is the baseline hazard, $exp\left(X_{i}^{⊤}\beta \right)$ is the relative risk function of $X_{i}$, and β denotes a p-vector of coefficients to be estimated. For each patient, we estimate the risk score, i.e., $X_{i}^{⊤}\beta$, a larger value of which implies a higher risk of mortality.

#### 4.1.3. Support Vector Machines.

Given the observed data, $\left\{\left(Y_{i},\delta_{i},X_{i}\right),i=1,\ldots ,n\right\}$, we estimate risk scores, $\psi^{⊤}X_{i}$ with $\psi \in R^{p}$, by solving

$$\begin{array}{r} \underset{\psi ,\xi }{min}\;\frac{1}{2}∥\psi {∥}^{2}+\gamma \sum_{i,j}\;\;v_{ij}\xi_{ij}\\ \text{ subject to }\psi^{⊤}\left(X_{j}-X_{i}\right)\geq -\xi_{ij}\\ \text{ and }\xi_{ij}\geq 0,i,j=1,\ldots ,n, \end{array}$$

where $v_{ij}=\delta_{i}I\left(Y_{i}<Y_{j}\right)$ is a comparability indicator for the ith and jth subjects, $\xi_{ij}$ is the pair-specific slack variable, and γ>0 is a regularization parameter. This version of the survival support vector machine is based on C-index (Harrell et al., 1982), which assesses the rank concordance between the predicted risk scores and survival times among comparable individuals, that is,

$$Pr\left({\text{ Score }}_{i}>{\text{ Score }}_{j}|T_{i}>T_{j}\right),$$

where $T_{i}$ and ${Score}_{i}$ are, respectively, the survival time and the risk score for subject i. In this setting, a larger value of the risk score implies a lower risk of mortality (Van Belle et al., 2007).

#### 4.1.4. Random Survival Forests and Survival Gradient Boosting.

Both methods (Hothorn et al., 2006; Ishwaran et al., 2008; Salerno & Li, 2022) aim to combine predictions from multiple survival trees. In random survival forests, we construct ‘B’ survival trees by resampling ‘B’ datasets of n observations with replacement and randomly selecting subsets of $p^{\prime }<p$ risk factors to train individual trees on. The log-rank test statistic is used as the splitting criterion (Shimokawa et al., 2015). With each tree grown on a different subset of $p^{\prime }$ predictors, we then combine the B trees into a random survival forest by averaging over the survival predictions for each tree (Ishwaran et al., 2011). In survival gradient boosting, we sequentially combine predictions from individual survival trees across ‘M’ steps, where M was tuned using cross-validation with respect to the C-index calculated on the training datasets (Hothorn et al., 2006). At the mth step, the predicted risk score is given by

$${ℱ}_{m}(X)={ℱ}_{m-1}(X)+w_{m}f_{m}(X),$$

where ${ℱ}_{m-1}(X)$ is the prediction from the previous step, $f_{m}(X)$ is a new prediction from a single tree in the current step, and $0<w_{m}\leq 1$ is the step size.

#### 4.1.5. Naive Ensemble Averaging.

To create an ensemble predictor, we combine the risk scores (after standardization as detailed in Section 4.1.6) generated by the four algorithms discussed. A basic method for forming an ensemble prediction for each subject would involve averaging the four risk scores. However, it is important to note that this approach assumes equal importance of individual learners in the construction of the ensemble risk score and that the pairwise correlations between these individual learners remain consistent.

#### 4.1.6. Weighted Ensemble Averaging.

A more principled approach is to weight the individual scores according to the information they provide, taking into account the covariance among the individual learners. Initially, we ensure that all scores generated by various algorithms align in the same direction: lower scores indicate a reduced mortality risk, and higher scores imply an elevated risk; if this alignment is not present, we reverse the sign of the scores. Subsequently, we employ a rank-based probit transformation to standardize these scores. This transformation maintains the interpretation that lower ranks correspond to lower mortality risk, while higher ranks signify higher mortality risk. Specifically, for n individual scores constructed by Algorithm a∈{1,…,A} (e.g.,  A=4 in our case), we convert them to percentile ranks, denoted by $r_{i}^{\left(a\right)},i=1,\ldots ,n$, and apply the probit transformation to obtain “standardized” risk scores, i.e.,

$$\phi_{i}^{\left(a\right)}=\Phi^{-1}\left\{\left(r_{i}^{\left(a\right)}-0.5\right)/n\right\},$$

where Φ{⋅} is the standard normal distribution function. We then construct a weighted ensemble prediction for each subject by the weighted average of the A standardized risk scores, i.e.,

$$\phi_{i}^{\left(e\right)}=\sum_{a=1}^{A}\;w^{\left(a\right)}\phi_{i}^{\left(a\right)}.$$

Ideally, we choose the weights $w^{\left(a\right)}$ by minimizing the mean squared error (Viana et al., 2009):

$$E\left\{{\left(\phi_{i}-\phi_{i}^{\left(e\right)}\right)}^{2}\right\}=w^{⊤}Cw,$$

where $w={\left(w^{\left(1\right)},\ldots ,w^{\left(A\right)}\right)}^{⊤}$. Here, $\phi_{i}$ denotes the ‘true’ risk score, calculated by applying the probit transformation to the rank based on the ‘true’ survival time of patient i=1,…,n. The matrix **C** is the A×A covariance matrix of the A individual prediction algorithms, where the (j,k)th element in the matrix is defined as $\left.c_{jk}=E\right]\left\{\left(\phi_{i}-\phi_{i}^{\left(j\right)}\right)\left(\phi_{i}-\phi_{i}^{\left(k\right)}\right)\right\}$ for 1≤j,k≤A. As we cannot observe the true risk scores for all patients due to censoring, we propose to estimate **C** by using the inverse probability weighted cross-validation errors. Denote its estimate by $\tilde{C}$, with the (j,k)th element defined as

$${\tilde{c}}_{jk}=\frac{1}{n}\sum_{i=1}^{n}\;\frac{\delta_{i}}{{\overset{ˆ}{\pi }}_{i}}\left(\phi_{i}-\phi_{-i}^{\left(j\right)}\right)\left(\phi_{i}-\phi_{-i}^{\left(k\right)}\right),$$

where $\phi_{-i}^{\left(a\right)}$ is the prediction of the ith subject obtained by applying Algorithm a to the data with the ith subject left out, as done in the ensemble literature for creating cross-validation errors (Acar & Rais-Rohani, 2009; Goel et al., 2007; Y. Lin et al., 2002; Meckesheimer et al., 2001). Here, ${\overset{ˆ}{\pi }}_{i}={\overset{ˆ}{S}}_{c}\left(Y_{i}|X_{i}\right)$, and ${\overset{ˆ}{S}}_{c}\left(\cdot |X_{i}\right)$ is the estimate of the survival function of the censoring time given $X_{i}$, i.e. $S_{c}\left(t|X_{i}\right)=Pr\left(C_{i}>t|X_{i}\right)$. It can be obtained by fitting a Cox proportional hazards model with the reversed censoring indicator, $1-\delta_{i}$. When the censoring time distribution is correctly specified, it can be shown that ${\tilde{c}}_{jk}$ consistently estimates $c_{jk}$ (Hothorn et al., 2006; Laan & Robins, 2003). As such, the problem can be formulated as

$$\underset{w}{min}\;\;w^{⊤}\tilde{C}w\;\text{ s.t. }\;1^{⊤}w=1,$$

and the solution can be obtained using Lagrange multipliers or, explicitly,

$$w=\frac{{\tilde{C}}^{-1}1}{1^{⊤}{\tilde{C}}^{-1}1}.$$

This approach might produce negative weights and weights exceeding one. To address this issue and ensure non-negativity, we employ only the diagonal elements of $\tilde{C}$ when computing the weights (Viana et al., 2009). Furthermore, these diagonal elements are known to be better approximated and more reliable compared to the off-diagonal ones (Viana et al., 2009). We applied this correction in the simulations in Section 5, and it yielded a superior performance compared to alternative methods, potentially confirming the efficacy of this approach.

### 4.2. C-Index for Prognostic Utility.

We first developed a series of predictive models by training the individual learners described above on a common set of demographic and clinical risk factors which we extracted from each patient’s electronic health record. Subsequently, we obtained predicted risk scores from these learners and combined them using the two ensemble averaging algorithms presented to derive combined risk scores for each patient. To determine whether X-ray imaging carried additional prognostic utility above and beyond the identified clinical risk factors, we repeated each procedure for the individual and ensemble learners, including both the clinical risk factors and screened radiomic features in the predictive models.

We assessed the predictive performance of each method using Harrell’s C-index. A higher C-index would indicate that the models with clinical and imaging features had a better performance in ranking subjects by predicted survival times as compared to the models with only clinical features (Longato et al., 2020). This would suggest that the radiomic features enhanced the model’s ability to differentiate between subjects experiencing events (deaths) at different times. To calculate the C-index, we partitioned the data into five folds, training each model on 80% of the data and testing on the remaining fold to compute the C-index. We repeated this process 100 times and reported the median as the estimate of the C-index. To gauge whether including radiomic features improved each model’s C-index, instead of reporting p-values which may not adequately account for the full spectrum of variations inherent in the estimation process (Greenland et al., 2016), we plotted the empirical distribution of C-index values across these 100 replications and reported the median and interquartile range (IQR) of this distribution.

### 4.3. Feature Importance.

To measure the importance of each risk factor across the various methods, we computed the decrease in C-index after removing the risk factor from the dataset (Breiman, 2001; Fisher et al., 2019). Risk factors with larger decreases in C-index were viewed as more important. Specifically, we utilized the permutation-based feature importance Algorithm 1 below (Molnar, 2020). We replicated this process on various patient subgroups to gain a better understanding of which patient groups would benefit most from our method. Specifically, we conducted subgroup analyses categorized by age (split at 65 years) and the number of existing comorbid conditions (split at the median of 9).


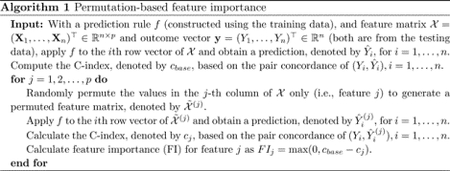


### 4.4. Adjusted Associations.

We fit a fully-adjusted Cox model with the final set of selected features to explore their connections with post-ICU mortality. Furthermore, a sensitivity analysis was conducted to scrutinize potential interactions between these risk factors and the prevailing COVID-19 variant during the infection period. This was undertaken to gauge the applicability of our findings across various phases of the pandemic.

## 5. Simulation Studies

We carried out a series of simulations to assess the performance of our proposed ensemble averaging method in comparison to the various machine learning approaches mentioned earlier.

### 5.1. Data Generation.

#### 5.1.1. Linear Log Hazards.

We designed our simulations to mimic the setting of the real data. We generated 22 covariates, $X_{i}={\left(X_{i,1},\ldots ,X_{i,22}\right)}^{⊤}\in R^{22},i=1,\ldots ,n$ independently across n subjects. from a multivariate Gaussian distribution, with a zero mean vector and a compound symmetric covariance matrix with unit variances and covariances equal to 0.2, i.e.,

$$X_{i}\sim {𝒩}_{22}\left\{0,\left[\begin{array}{cccc} 1 & 0.2 & \ldots & 0.2\\ \vdots & \vdots & \ddots & \vdots \\ 0.2 & 0.2 & \ldots & 1 \end{array}\right]\right\}.$$

We assume that among them, 12 are related to the true survival time. We also assume that censoring time can be covariate dependent so that 9 covariates are related to the censoring time. There are 3 covariates that are related to both survival and censoring times. In our later real data analysis, 13 variables were found to be relevant to survival. We simulated the true survival time for each observation, $T_{i}$, from an exponential distribution with a hazard of

$$\lambda \left(X_{i}\right)=\mu_{T}\times exp\left\{X_{i,1}\beta_{1}+\cdots +X_{i,12}\beta_{12}\right\},$$

where the log hazard is linear in $X_{i}$. Moreover, we independently generated censoring times, $C_{i}$, from an exponential distribution with a hazard of

$$\lambda_{c}\left(X_{i}\right)=\mu_{C}\times exp\left\{X_{i,10}\alpha_{1}+\cdots +X_{i,18}\alpha_{9}\right\}.$$

The α and β coefficients were generated from uniform distribution, 𝒰(−1,1). We selected values for $\mu_{T}$ and $\mu_{C}$ to introduce varying levels of approximate censoring rates, specifically targeting rates of 40%, 60%, 70%, and 80%. We aimed to evaluate the performance of each prediction approach across these four censoring scenarios. Within each scenario, we generated a total of 100 independent datasets, with each dataset comprising n=2,300 observations. These settings were chosen to closely resemble our real data, taking into account both the sample size and the desired approximate censoring rates. Within each dataset, 80% of the observations were allocated for the purpose of training and fitting various models, while the remaining 20% were held aside as testing data for model evaluation. To assess the effectiveness of each model, we employed the C-index metric to evaluate the predictions made on the testing data.

#### 5.1.2. Nonlinear Log Hazards.

Our setup was similar to the linear setting, except that we simulated the survival and censoring times from hazards that are nonlinear in covariates. Specifically, we simulated survival times, $T_{i}$, from an exponential distribution with a hazard of

$$\lambda \left(X_{i}\right)=\mu_{T}\times \text{exp}\{0.3\text{exp}\left(X_{i,1}-X_{i,2}\right)-0.3\text{log}\left\{{\left(X_{i,3}+X_{i,4}\right)}^{2}\right\}+0.25\text{sin}\left(X_{i,5}X_{i,6}\right)\;-0.2{\left(X_{i,7}-X_{i,8}+X_{i,9}\right)}^{2}-0.2\left|X_{i,10}-X_{i,11}+X_{i,12}\right|\},$$

where the log hazard is nonlinear in $X_{i}$. We then independently generated censoring times, $C_{i}$, from an exponential distribution with a hazard of

$$\lambda_{c}\left(X_{i}\right)=\mu_{C}\times \text{exp}\{0.15\text{sin}\left(X_{i,10}-X_{i,11}+X_{i,12}\right)-0.05{\left(X_{i,13}-X_{i,14}+X_{i,15}\right)}^{2}\;+0.05\text{exp}\left(X_{i,16}-X_{i,17}+X_{i,18}\right)\}.$$

We again selected values for $\mu_{T}$ and $\mu_{C}$ with the aim of introducing varying levels of approximate censoring rates, specifically targeting rates of 40%, 60%, 70%, and 80%. We followed the identical procedure as detailed in the preceding subsection to evaluate the performance of our prediction algorithm. Our objective was to assess how well the algorithm performed when the assumptions of the linear log hazard model were not met.

### 5.2. Simulation Results.

Table 1 presents the outcomes of the simulations where the datagenerating mechanism adheres to a linear log hazard model. In this scenario, it is evident that all prediction models exhibit commendable performance, with the Cox model with linear log hazards consistently outperforming the others across all levels of censoring rates. Specifically, the median C-index for the Cox model ranges from 85.9% (at a 40% censoring rate) to 90.1% (80% censoring), and survival support vector machines (SVM) follow closely in performance (85.8% to 89.8%). The proposed weighted ensembling approach exhibits competitive results across all censoring rates, yielding C-indices (85.6% to 89.5%) close to those achieved by the Cox model and survival SVM. For context, note that there are 2,643,850 unique pairs of observations in each dataset. Therefore, a 1% increase in C-index corresponds to 26,000 more patient pairs being correctly ranked in terms of their mortality risk. It is worth noting that as the censoring rate increases, the predictive performance of all methods shows improvement, as the C-index may increase when focusing on discrimination of earlier events or among higher risk patients (Longato et al., 2020).

Table 2 presents the simulation results for the scenario where the data were generated with the log hazards nonlinear in the predictors, and all methods demonstrate a decline in prediction as they grapple with the increasingly complex nature of the risk relationship. In this challenging context, the weighted ensemble averaging method consistently outperforms all other competing methods, achieving the highest median C-index across all levels of censoring rates (73.1% at 40% censoring to 78.9% at 80% censoring). Furthermore, it showcases numerical stability with the narrowest IQRs when compared to all other methods. Conversely, when the linearity assumption breaks down, the Cox model exhibits the poorest performance in terms of the C-index, ranging from 65.6% at 40% censoring to 72.5% at 80% censoring. We again note the upward trend of the predictive performance of the methods as censoring rates increase.

### 5.3. Sensitivity Analysis for Missing Data.

In a sensitivity analysis, we conducted additional simulations to assess the impact of the mean imputation strategy on each model’s performance, including our proposed weighted ensemble. We simulated data as described above, but we additionally simulated missingness under the assumption that the data are missing at random (MAR) (Rubin, 1976). We assessed the performance of the mean imputation by varying the percentage of missingness for each covariate from the complete case (0%) to 10% and 20%, corresponding respectively to the average and maximal missingness percentage among the included variables in the real data analysis. The findings indicate that the conclusions drawn in scenarios with the complete data remain valid when there are missing data, even when imputed using means. This hints at the robustness of the predictive models used. However, the efficacy of predictive models based on mean imputation decreases as the proportion of missing data rises. This decline is attributed to the fact that covariates were generated from a multivariate normal distribution with non-zero correlations and mean imputation might overlook such information. For additional details, see Appendix B.

## 6. COVID-19 ICU Data Analysis Results

### 6.1. Outcome Distribution.

**Figure 4.**
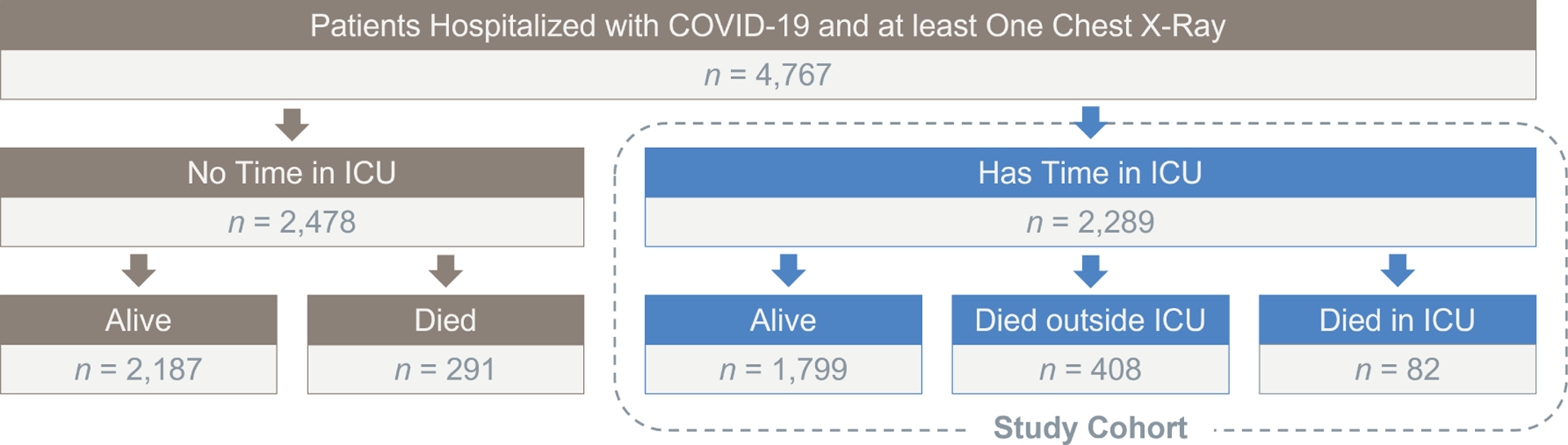
Flowchart of outcomes and derivation of our study population (n=2,289).

Figure 4 illustrates the derivation of the study population and provides a breakdown of the initial population based on their ICU and mortality outcomes. Notably, it reveals that 48% of admitted patients required ICU escalation at some point during their hospital stay, making them the focus of our study. Among these patients, 21% died while in the ICU, in contrast to 12% of patients who did not necessitate ICU admission. Among those who succumbed in the ICU, 3% did so during their initial ICU encounter, while 18% passed away after being transferred to a lower level of care or discharged.

### 6.2. Characteristics of the Study Population.

Out of 2,289 patients in our study, 1,528 (66.8%) were diagnosed during the dominance of the original variant, 320 (14.0%) during the Alpha variant, and 441 (19.2%) during the Delta variant in Southeast Michigan. The median age was 61 years [Interquartile Range (IQR): 29], which differed by wave of the pandemic; it was higher among those infected earlier (62 years; IQR: 26) versus during the Alpha (59 years; IQR: 36) and Delta (59 years; IQR: 34) waves. Further, self-reported race differed significantly by wave, with a higher proportion of patients identifying as White in later waves of the pandemic (Original: 67%, Alpha: 72%, Delta: 80%) than patients of color. Noticeably, patients diagnosed and admitted to the ICU with the original variant tended to have a higher comorbidity burden on average than patients in the Alpha and Delta waves. Moreover, the vast majority of patients (1,914; 84%) were not vaccinated at the time of ICU escalation, while 291 (13%) were fully vaccinated, and a small minority (84; 3.7%) were partially vaccinated, with these proportions diverging in later waves. Full descriptive characteristics for this patient population are given in Appendix A, Table A2.

**Figure 5.**
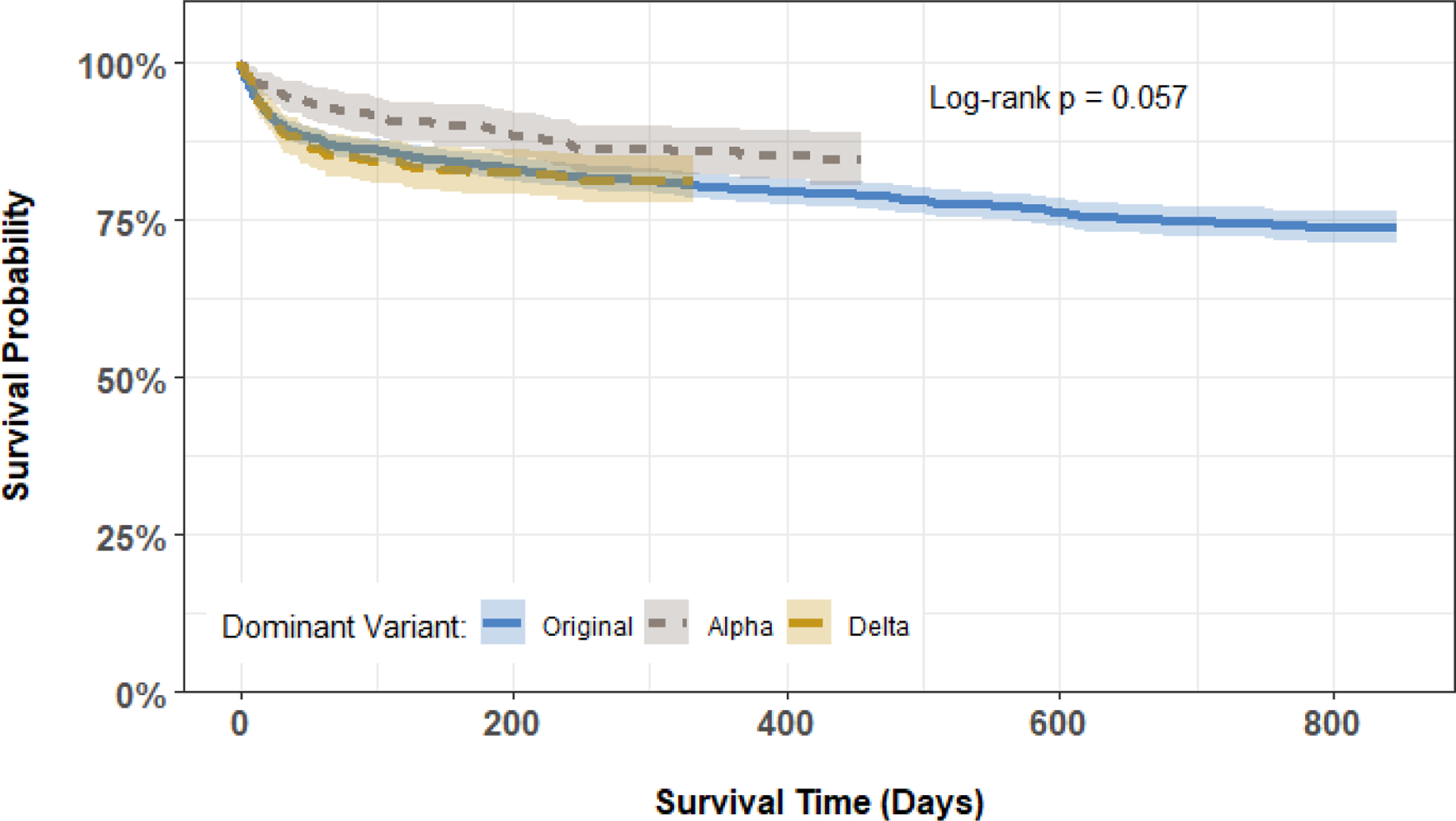
Kaplan-Meier curves comparing post-ICU survival among the 2,289 patients in the study cohort, stratified by dominant COVID-19 variant at diagnosis.

Kaplan-Meier estimated survival curves for post-ICU mortality are given in Figure 5, stratified by dominant variant. Marginally, patients diagnosed during the Alpha wave had slightly better survival than patients diagnosed during the Original or Delta waves; however, these unadjusted differences were not statistically significant.

### 6.3. C-Index for Prognostic Utility.

**Figure 6.**
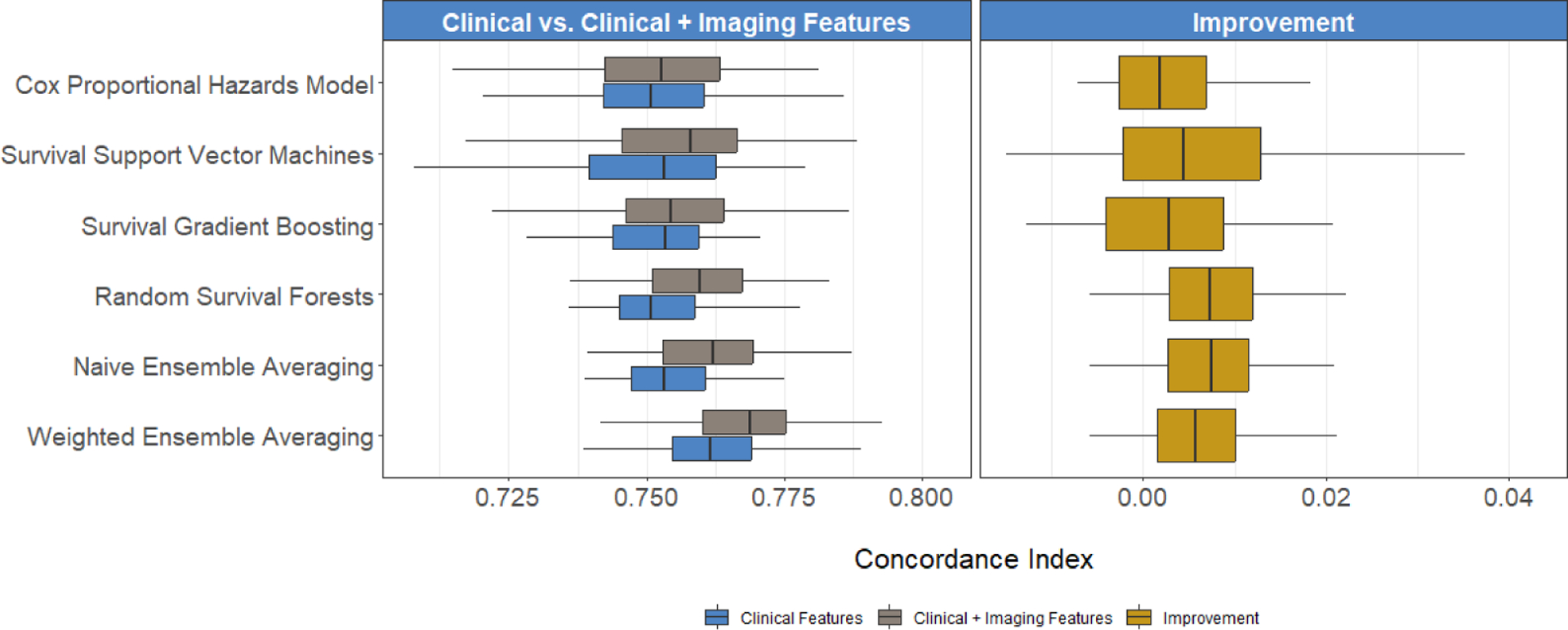
Comparisons of C-index when using clinical and clinical + imaging-derived risk factors, obtained by six machine learning algorithms. Boxplots report the distribution of the C-index across 100 training and testing experiments for the clinical versus clinical + imaging models (left) and the distribution of improvement in the C-index (right).

**Figure 7.**
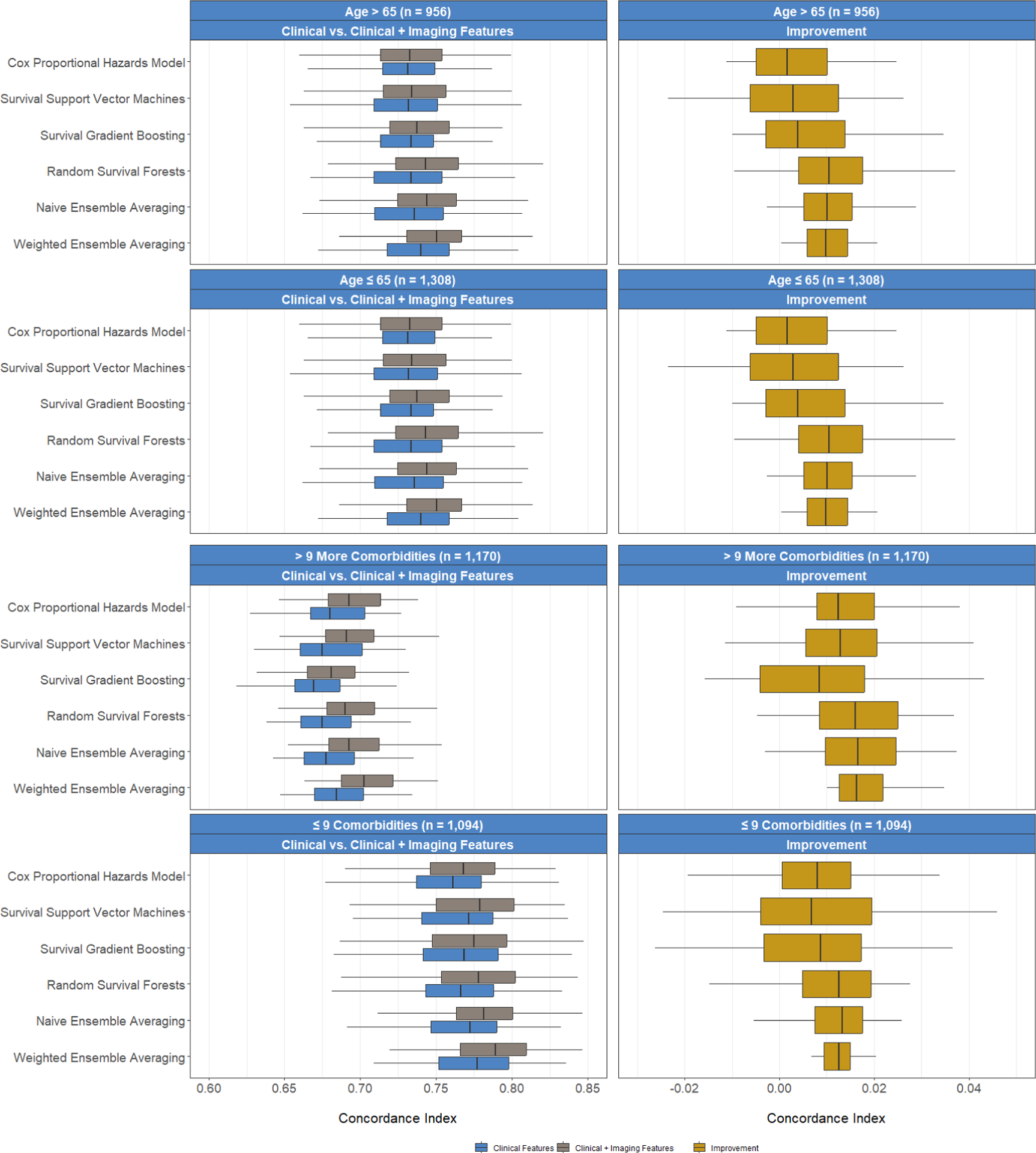
Prediction performance in C-index of different algorithms comparing (1) patients 65 years or younger versus older than 65 years, and (2) patients with ≤ 9 (median) versus > 9 comorbidities. Plot rows depict different subsets of patients, while plot columns show the C-index empirical distributions for the clinical versus clinical + imaging feature models (left) and the distribution of improvement in the C-index (right).

**Figure 8.**
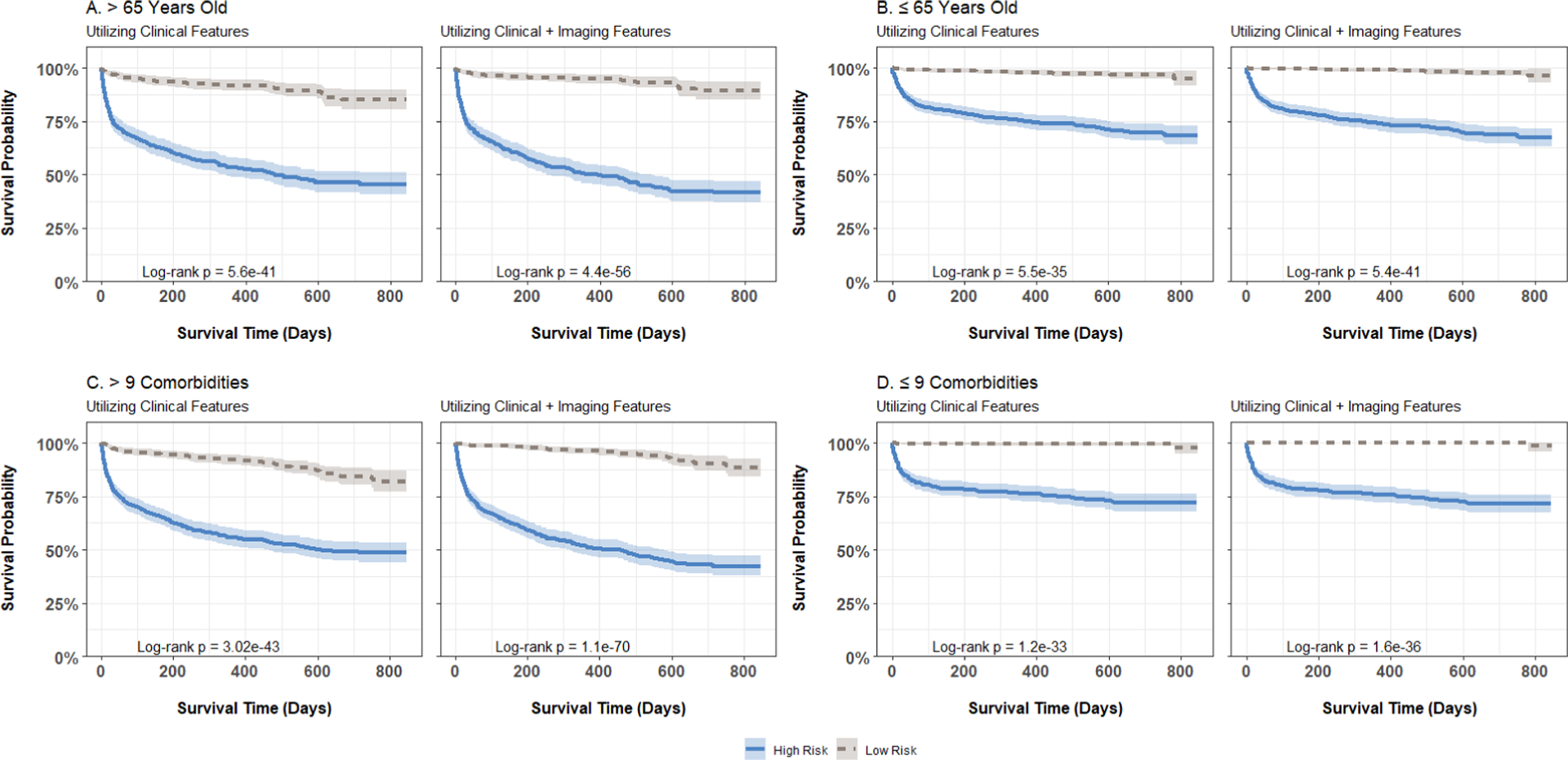
Kaplan-Meier curves for post-ICU escalation mortality, stratified by patient age and risk group (defined by median risk score), computed using weighted ensemble averaging models incorporating clinical or clinical plus imaging features within each age and comorbidity burden category: (A) age > 65, (b) age ≤ 65, (c) comorbidities > 9, (d) comorbidities ≤ 9. High-risk groups are represented by solid lines, while low-risk groups are depicted with dashed lines.

We first built predictive models using only the clinical and demographic risk factors derived from each patient’s EHR. Across the six methods under consideration, we calculated the median C-indices ranging from 75.1% to 75.4% among the individual learners, 75.3% with naive ensemble averaging, and 76.2% with our weighted ensemble averaging (Figure 6). With the addition of the screened radiomic features, we observed an increase in C-index for survival support vector machines [median (IQR) C-index of 75.3 (2.3) versus 75.8 (2.0)], random survival forests [75.1 (1.4) versus 76.0 (1.6)], naive ensemble averaging [75.3 (1.4) versus 76.2 (1.6)], and weighted ensemble averaging [76.2 (1.4) versus 76.9 (1.5)] approaches. Across all models and feature subsets, the weighted ensemble averaging with both clinical and radomic features yielded the highest C-index (76.9%). In subgroup analyses, we found that predictions on younger (≤ 65 years old) and healthier (≤ 9 comorbidities) patients were more accurate than those on older (> 65 years) patients and those with higher comorbidity burden (> 9 comorbidities). However, including imaging data resulted in greater improvement in prediction performance in all subgroups compared to models trained solely on clinical and demographic risk factors. The improvement realized with the addition of radiomic features was higher, on average, among older and sicker patients across all predictive models (Figure 7). Finally, we plotted Kaplan-Meier survival estimates for each patient subgroup, stratified by high- versus low-predicted risk scores from our ensemble-averaged model. Our results demonstrate significant differences in survival between high- and low-risk patients across all subgroup comparisons (log-rank p-values < 0.0001), with greater differences among older and sicker patients (Figure 8).

### 6.4. Feature Importance.

**Figure 9.**
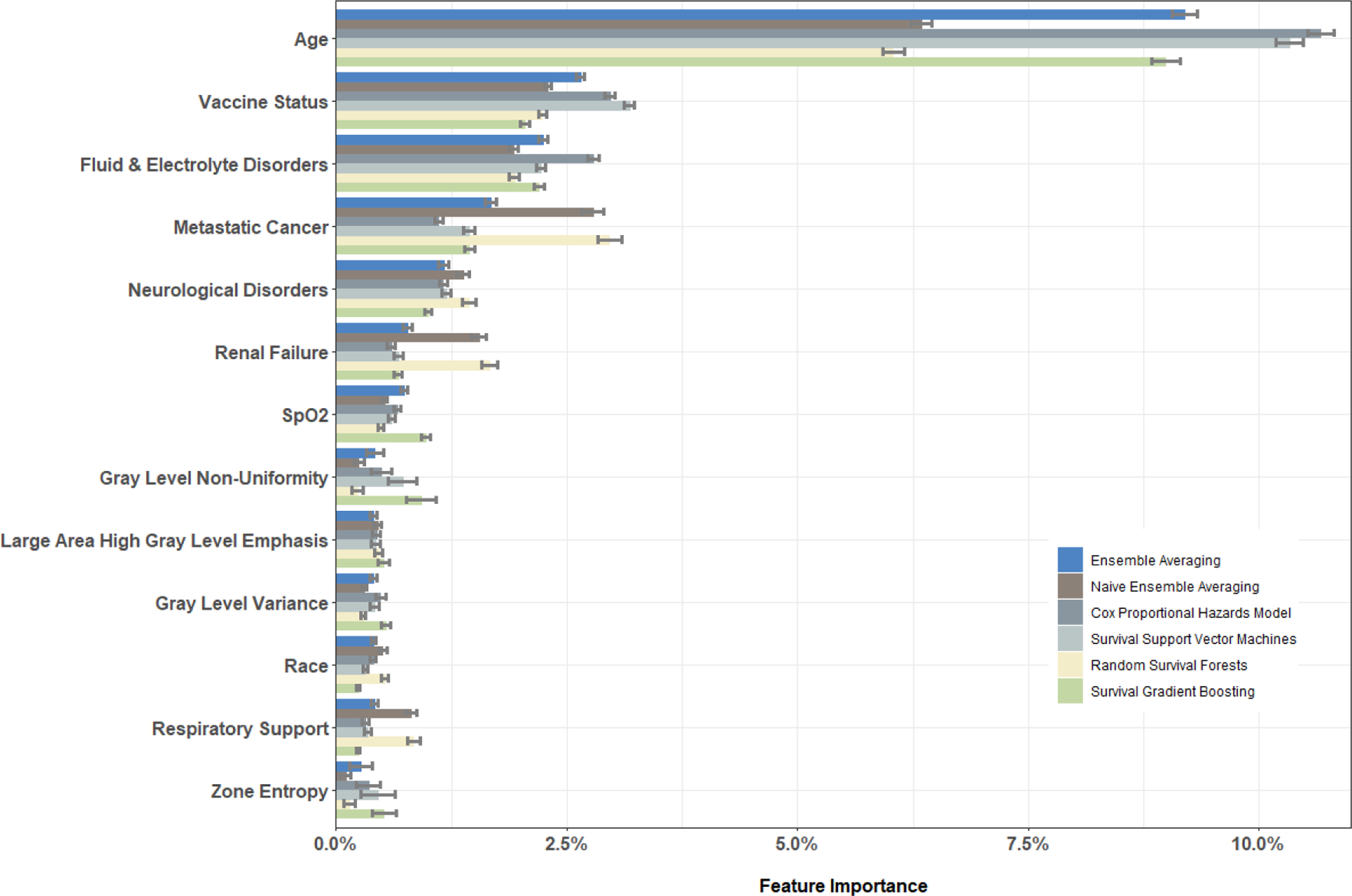
Feature importance for selected clinical and imaging features and the associated standard deviations (error bars).

Figure 9 reports the values of feature importance (as defined in Section 4.3) for the set of selected features. Age was the most important predictor of post-ICU mortality across all methods, followed by vaccination status. Further, we found that certain prevalent comorbidity conditions such as indications of fluid and electrolyte disorders, metastatic cancers, neurological disorders, renal failure, physiologic measurements such as oxygen saturation (SpO2), need for respiratory support, and a patient’s race were predictive of mortality to a lesser extent. Important imaging texture features included gray-level non-uniformity and gray level variance, measures of the variability pixel intensity values in the image, large area high gray level emphasis, a measure of the proportion of the image with larger sized zones of higher gray-level values, zone entropy, a measure of heterogeneity in the texture patterns (Zwanenburg et al., 2020).

### 6.5. Associations between Selected Risk Factors and Mortality.

We considered the selected risk factors in a fully-adjusted Cox proportional hazards model. Table 3 presents the estimated hazard ratios (HR) and 95% confidence intervals (CI), showing that older age (HR: 1.03; CI: 1.03–1.04), indications of fluid and electrolyte disorders (HR: 3.12; CI: 2.21–4.40), metastatic cancers (HR: 1.51; CI: 1.25–1.83), neurological disorders (HR: 1.60; CI: 1.33–1.92), renal failure (HR: 1.32; CI: 1.09–1.61), and need for respiratory support (HR: 1.39; CI: 1.12–1.72) were significantly associated with higher post-ICU mortality, while higher oxygen saturation (HR: 0.93, CI: 0.90–0.97) was significantly associated with lower mortality. We also found that being either partially (HR: 0.45; CI: 0.27–0.75) or fully (HR: 0.32; CI: 0.22–0.45) vaccinated was also significantly associated with lower mortality in a seemingly dose-response relationship. We applied a Cox model including interactions between all other selected risk factors and the dominant variant at diagnosis; see Table 3. To assess how the effects of risk factors differed across the different waves of the pandemic, we considered the significant interactions with each main effect (where the original variant served as the reference group). The estimated associations for each risk factor during each wave of the pandemic were largely consistent, except for vaccination status during the Alpha wave, where the effect of vaccination was weaker in this wave.

## 7. Discussion

### 7.1. What We Have Addressed.

The COVID-19 pandemic has led to a proliferation of machine learning tools aimed at predicting increasingly severe outcomes, such as infection, hospitalization, ICU escalation, and mortality. Early in the pandemic, accurate risk stratification was crucial to effectively allocate resources (F.-Y. Cheng et al., 2020; Hartman et al., 2020; Knight et al., 2020; Van Singer et al., 2021). Given the severity of COVID-19, understanding post-ICU outcomes is of particular interest as patients may experience lasting pulmonary and neurological morbidity. This study aimed to explore the prognostic value of radiomic features among COVID-19 patients who required ICU-level care. Our findings revealed that age, vaccination status, fluid and electrolyte disorders, metastatic cancers, neurological disorders, oxygen saturation, and race were important risk factors. In terms of imaging features, pixel heterogeneity measures proved significant. We observed improvements in performance across four individual prediction models and an ensemble predictor when including imaging data in addition to clinical risk factors. Furthermore, the improvement with the inclusion of radiomic features was higher among older and sicker patients.

Our work exemplified a valuable experience of leveraging the vast resources available through DataDirect and the Precision Health Initiative to identify important radiomic features for predicting COVID-19 survival among a highly vulnerable subset of patients with the most severe disease. By integrating electronic health records and chest X-ray databases, we have created a framework that allows for convenient linkage between imaging studies and essential clinical information. Our standardized workflow for image pre-processing, feature selection, and predictive modeling ensures reproducibility of results. Furthermore, our findings were consistent with the growing literature on ICU outcomes for COVID-19 patients. For example, a post-ICU mortality rate of 21.57% was reported in the first wave of the pandemic (Ramani et al., 2021), a rate similar to what we observed (21.41%); like our study, other authors also identified age, gender, sequential organ failure assessment score, Charlson Comorbidity Index score, Palliative Performance Score, and need for respiratory support as risk significant factors for COVID-19 mortality (Lorenzoni et al., 2021).

Our image analysis results were also consistent with several recent studies that explored the use of COVID-19 chest X-ray images as COVID-19 predictors. For example, a previous study identified 51 radiomic features associated with COVID-19, six of which were predictive of short-term mortality, including low gray-level emphasis and size zone matrix non-uniformity (Ferreira Junior et al., 2021), which coincided with our findings. A deep learning algorithm was proposed to extract features that correlated with radiologic labels predicting worsening disease trajectory and the need for mechanical ventilation, and AUCs were reported to range from 0.64 to 0.74, and 0.81 in an open-access dataset (Gourdeau et al., 2022a; Gourdeau et al., 2022b), which were close to our results as well; inclusion of imaging data was found to improve prediction, with an AUC of 0.70 and an accuracy of 0.69, compared to an AUC of 0.65 and an accuracy of 0.66 using clinical data alone (J. Cheng et al., 2022), which corroborated with our findings.

### 7.2. Risk Factors of Potential Interest.

By and far, the most important risk factor across all methods was age (Ji et al., 2020; Richardson et al., 2020; Weng et al., 2020). Further sub-group analysis revealed that our methods had higher predictive utility among patients 65 years of age or younger; however, the subgroup containing patients over 65 years saw the most improvement in prognostication with the additional information from their chest X-rays. This is consistent with our previous work, which considered outcomes of varying severity, including inpatient mortality among all hospitalized patients (Salerno, Sun, et al., 2021; Sun et al., 2022). Recent studies have supported these results, including a systematic review and meta analysis, which showed that older age was significantly associated with disease severity, as well as six prognostic endpoints (Fang et al., 2020; Figliozzi et al., 2020; Güllü et al., 2021).

Vaccination status was another factor that was shown to be predictive across all methods explored in this analysis, with partial or full vaccination having a statistically significant protective effect with a dose-response relationship in fully-adjusted models for associations. We note that this result has mixed support in the recent literature. Many studies have confirmed that COVID-19 vaccination is efficacious in reducing rates of endpoints such as severe disease, hospital admission, ICU escalation, or need for respiratory support/mechanical ventilation; however, with respect to post-ICU mortality, these studies failed to find statistically significant differences in outcomes (AlQahtani et al., 2022; Freund et al., 2022; Grasselli et al., 2022). One recent study found differences in mortality rates by patient vaccination status, specifically among non-immunocompromised patients as opposed to those patients who were identified as being immunocompromised (Singson et al., 2022). Overall, the vaccination rate in this patient population was low, particularly in later waves of the pandemic, lending additional evidence to the underuse of vaccines in populations with severe diseases.

Additional comorbid conditions, including metastatic cancers, neurologic, and fluid and electrolyte disorders, were also found to be predictive of post-ICU mortality, as well as associated with this outcome in adjusted models. It is well known that patients who are immunocompromised, particularly those with late-stage cancers, are more likely to experience severe complications from COVID-19, such as acute respiratory distress syndrome, liver injury, myocardial injury, and renal insufficiency, leading to worsened outcomes (Han et al., 2022; Yang et al., 2020; Zhang et al., 2021). Beyond the direct impact of COVID-19 infection, indirect effects of the pandemic such as disruptions to cancer diagnosis, management, and surgical intervention have also been shown to impact years of life lost and attributable deaths in these vulnerable populations, necessitating the development of strategies for resource allocation and care management early on (Hartman et al., 2020; Sud et al., 2020). Lastly, the presence of fluid and electrolyte disorders on ICU escalation implies an increased severity of a patient’s disease course, especially given what is known about COVID-19 involvement across multiple organ systems (Chiam et al., 2021; De Carvalho et al., 2021; Nahkuri et al., 2021; Pourfridoni et al., 2021).

We found oxygen saturation to be the only physiologic measurement under our consideration that was predictive of mortality. Oxygen saturation is known to be indicative of worsening outcomes for patients with COVID-19, especially as a precursor to acute respiratory distress syndrome and mortality (Bhatraju et al., 2020; Matthay et al., 2020; Zhao et al., 2020). Median oxygen saturation was 95.57% in our patient population. This is notably low, given that roughly 30% of patients were receiving supplemental oxygen support prior to ICU escalation, and thus may be reflective of progressive hypoxia or future respiratory decompensation.

Important radiomic features included gray level non-uniformity, zone entropy, gray level variance, and large area high gray level emphasis, which characterize the heterogeneity in the texture patterns and variability of pixel intensity values on chest X-ray (Zwanenburg et al., 2020). Our previous work reported similar findings among hospitalized patients with COVID-19. Namely, we found that zone entropy and dependence non-uniformity, measures of feature heterogeneity, were predictive of in-hospital mortality, in addition to median pixel intensity and large dependence high gray level emphasis (Sun et al., 2022). Similar results were reported (Varghese et al., 2021).

### 7.3. Considerations on Using Image Data for COVID-19 ICU Outcome Prediction.

In our study, a 2% increase in the C-index, resulting from including radiomic features, translates to correctly ranking approximately 50,000 more patient pairs regarding their mortality risk out of a total of 2,618,616 possible pairs. This is meaningful, especially given the clinical complexity of these patients, many of whom suffer from multi-organ failure and multiple comorbidities. Our findings suggest that the greatest improvement in prognostic utility is among older and sicker patients, typically challenging to risk-stratify in acute care settings. Importantly, the use of imaging alongside clinical indicators for prognostication in COVID-19 acute care settings is relatively novel. X-ray images in the COVID-19 ICU population are primarily used for specific medical decisions, like dosing diuretics, adjusting ventilator settings, or placing endotracheal tubes and central venous catheters. However, our data indicate that X-rays in the COVID-19 ICU population can be meaningfully used for risk stratification and prognostication, which could play a crucial role in informing the overall course of a patient’s ICU stay. Nevertheless, X-rays introduce additional burdens for patients, physicians, and higher medical costs. Identifying subgroups where these imaging features are valuable for risk prediction can guide clinical practice. Furthermore, it is worth noting that multi-modal data and the integration of radiomic data with clinical risk factors are not commonly utilized, especially in the context of COVID-19. This patient population presents unique challenges, as the presence of significant findings on X-rays does not always correlate with a poor outcome. For example, some younger, healthier patients with bilateral infiltrates on chest X-rays may not require hospital-level care (Long et al., 2020). Knowing when and for whom X-rays are useful is essential information to guide clinical practice.

We discussed the use of machine learning for predicting COVID-19 outcomes and constructing a dependable ensemble risk score. Our main objective was to evaluate the added prognostic value of imaging features in clinical prediction models. Our findings demonstrated enhancements in predictive accuracy, especially within specific patient subgroups. In our approach, we summarized data from chest X-ray images using texture features, which quantify pixel intensity distributions and heterogeneity through various derived metrics. Our aim was to offer a statistically rigorous, interpretable, and replicable method for integrating imaging information into predictive models. Other potential analytic choices, as discussed above, include radiologist-derived severity scores and deep learning of the raw images (J. Cheng et al., 2022; Gourdeau et al., 2022a; Gourdeau et al., 2022b). While these approaches may be subject to certain biases, in future work, we believe it is necessary to compare various approaches to better understand their relative strengths and pitfalls. Other approaches, such as methods developed for image segmentation, are promising, but these approaches often rely on supervised learning, meaning that segmentation maps are necessary to train the models. As no segmentation maps exist for COVID-19 images, utilizing this information effectively is still an open problem.

Our framework enabled us to leverage survival data, with a relatively long observation period, to identify features that were most strongly associated with patient outcomes. Throughout our workflow, we fully utilized the time-to-event data as the outcomes for feature screening and selection, predictive modeling, and the final associative model. This is in contrast to many predictive studies that use dichotomous outcomes such as death (yes/no), without considering the duration of followup or the possibility of censoring. However, there is room for future development and improvement by incorporating longitudinal clinical and X-ray information in our prediction model. This could provide a better understanding of how patient survival experience changes throughout the course of the disease. Further, we performed marginal screening on each feature, with a significance threshold of α=0.05 for retaining features. Alternative screening approaches, as compared to marginal screening, could be to conduct Cox regression conditional on a small number of principal components or other lower-dimensional representation of the data (Liu et al., 2017), or to use the false discovery rate to control the number of retained features (Benjamini & Hochberg, 1995).

Another area of consideration in this study was whether the model trained on current data, which included original, Alpha, and Delta variants, could be easily applied in the future as new variants may evolve and therapeutic practices change. We considered the dominant variant at diagnosis and a patient’s vaccination status as proxies for how the pandemic has evolved. Our model showed a degree of robustness to differences in the dominant variant when we explored our selected features in associative models.

We primarily focused on portable chest X-ray as the imaging modality due to its convenience and efficacy in triaging emergent cases. However, other imaging platforms, such as chest CT scans, may provide higher quality imaging, especially in settings where patients remain for extended periods. It would be valuable to explore the differences in information that can be obtained from these imaging modalities and incorporate these insights into our predictive models. Doing so can help us better comprehend the underlying mechanisms of disease progression and ultimately enhance patient outcomes, as well as lead to future work extending these methods to analyze the use of imaging features to improve prediction of treatment responses, furthering our understanding of imaging-guided therapy for COVID-19 (Bard, 2021).

Finally, we recommend the use of an ensemble learning approach to improve risk prediction. By integrating risk predictions from established and effective machine learning techniques, ensembling enables us to harness more information to create more precise predictions. Our approach to weighted ensembling involved optimizing the ensembling weights and considering the shared variability of the individual risk scores. However, other ensembling approaches such as Super Learner can be used, which weights each algorithm in the ensemble by its cross-validation performance (Van der Laan et al., 2007). Using a ‘smart’ ensembling approach, in general, could provide valuable insights for clinical decision-making and aid clinicians in identifying patients with a higher risk of mortality following escalation to intensive care.

### 7.4. Limitations.

This is a single-center study at Michigan Medicine. Enhancing generalizability would require external validation, including predictive modeling on an independent validation set. Additionally, our data predates the dominance of the Omicron variant due to database update delays, warranting a future analysis on an Omicron cohort for the robustness of results. Patients transferred from out of state, especially those needing higher care levels, may lack accurate immunization records, potentially weakening vaccine effects. Differentiating between vaccine types and booster doses could enhance our understanding of vaccination’s prognostic value. Our sensitivity analysis indicates that the performance of predictive models based on mean imputation declines with an increasing proportion of missing data. Exploring alternative multiple imputation techniques, particularly those tailored for machine learning, may enhance the prognostic utility of the proposed approach, particularly in scenarios where data are missing at random. (Lo et al., 2019; Rubin & Schenker, 1986). Finally, extending these methods to handle longitudinal data could better quantify changes in a patient’s clinical course, informing therapeutic decisions.

### 7.5. Conclusions.

This work presents an analytic workflow for combining clinical, socio-demographic, and radiomic risk factors for COVID-19 mortality after escalation to an intensive care setting. Our findings demonstrate the additional prognostic benefits of incorporating imaging information into various prediction models, particularly among certain vulnerable patient sub-populations. These results are supported by a growing body of literature and our previous experience working with data on COVID-19 patients at Michigan Medicine, as well as the resources available to us through DataDirect. The DataDirect COVID-19 clinical data and X-ray database is a crucial part of a new precision health initiative established in Michigan Medicine during the pandemic, and its infrastructure has provided an invaluable platform for facilitating our work. Future studies that leverage detailed patient information in EHRs, such as patient demographics, comorbidity conditions, physiological measurements, treatment history, and temporal relationships between infection and subsequent outcomes, will continue to provide insights into the lingering impact of the pandemic, informing the long-term management of patients recovering from COVID-19.

## Figures and Tables

**Table 1.** Comparisons of median C-indices of six machine learning algorithms across varying censoring rates (40% to 80%) under a linear log hazard model using complete data. The table displays median C-index values from 100 experiments, along with their interquartile ranges.

	Censoring Rate			
	40%	60%	70%	80%
Cox Model with Linear Log Hazards	85.9 (1.6)	87.9 (1.6)	88.0 (1.8)	90.1 (1.5)
Survival Support Vector Machines	85.8 (1.6)	87.8 (1.6)	87.8 (1.9)	89.8 (1.4)
Survival Gradient Boosting	84.6 (1.7)	86.3 (1.8)	86.1 (2.1)	88.0 (1.9)
Random Survival Forests	83.1 (2.0)	84.8 (1.9)	85.3 (2.3)	86.9 (2.3)
Naive Ensemble Averaging	83.1 (2.0)	85.0 (1.9)	85.5 (2.3)	87.3 (2.1)
Weighted Ensemble Averaging	85.6 (1.4)	87.4 (1.7)	87.4 (1.8)	89.5 (1.5)

**Table 2.** Comparisons of median C-indices of six machine learning algorithms across varying censoring rates (40% to 80%) under a nonlinear log hazard model using complete data. The table displays median C-index values from 100 experiments, along with their interquartile ranges.

	Censoring Rate			
	40%	60%	70%	80%
Cox Model with Linear Log Hazards	65.6 (2.0)	67.8 (3.0)	69.0 (2.9)	72.5 (3.1)
Survival Support Vector Machines	73.0 (1.7)	74.6 (1.4)	76.3 (1.8)	78.7 (2.4)
Survival Gradient Boosting	72.6 (1.3)	74.2 (1.6)	75.7 (2.1)	78.0 (2.3)
Random Survival Forests	71.5 (1.5)	73.3 (1.6)	75.2 (2.0)	77.5 (1.9)
Naive Ensemble Averaging	71.5 (1.5)	73.4 (1.6)	75.3 (2.0)	77.6 (1.9)
Weighted Ensemble Averaging	73.1 (1.1)	74.8 (1.4)	76.6 (1.6)	78.9 (1.6)

**Table 3.** Adjusted associations between identified risk factors and mortality after the ICU admission, among the 2,289 patients with COVID-19 and stratified by dominant variant at diagnosis.

	A. Without Interactions			B. With Interactions by Dominant Variant				
	All Variants		Original		Alpha		Delta	
Characteristic	HR	CI	HR	CI	HR	CI	HR	CI
Age	1.03	(1.03, 1.04)	1.04	(1.03, 1.05)	1.04	(1.03, 1.05)	1.02	(1.01, 1.03)
Fluid & Electrolyte DiD	2.77	(1.95, 3.94)	3.19	(2.04, 4.99)	3.19	(2.04, 4.99)	1.60	(0.82, 3.13)
Vaccination Status								
Not Vaccinated	-	-	-	-	-	-	-	-
Partially Vaccinated	0.44	(0.26, 0.75)	0.35	(0.18, 0.65)	1.66	(0.48, 5.76)	0.35	(0.18, 0.65)
Fully Vaccinated	0.32	(0.22, 0.46)	0.29	(0.19, 0.45)	0.75	(0.31, 1.78)	0.29	(0.19, 0.45)
Metastatic Cancer	1.42	(1.17, 1.73)	1.43	(1.14, 1.80)	1.43	(1.14, 1.80)	1.43	(1.14, 1.80)
Neurological DiD	1.49	(1.24, 1.80)	1.43	(1.15, 1.78)	1.43	(1.15, 1.78)	1.43	(1.15, 1.78)
Renal Failure	1.32	(1.09, 1.61)	1.12	(0.89, 1.41)	1.12	(0.89, 1.41)	2.59	(1.58, 4.25)
Oxygen Saturation	0.93	(0.89, 0.96)	0.94	(0.89, 0.98)	0.94	(0.89, 0.98)	0.94	(0.89, 0.98)
Respiratory Support								
No	-	-	-	-	-	-	-	-
Yes	1.39	(1.12, 1.72)	1.34	(1.04, 1.73)	1.34	(1.04, 1.73)	1.34	(1.04, 1.73)
Unknown	1.06	(0.79, 1.42)	1.16	(0.83, 1.63)	1.16	(0.83, 1.63)	1.16	(0.83, 1.63)
Race								
White	-	-	-	-	-	-	-	-
Black	0.98	(0.77, 1.25)	0.94	(0.71, 1.24)	0.94	(0.71, 1.24)	0.94	(0.71, 1.24)
Other/Unknown	1.45	(1.11, 1.90)	1.40	(1.03, 1.90)	4.35	(1.94, 9.76)	1.40	(1.03, 1.90)
Gray Level Nonuniformity	1.02	(0.88, 1.18)	1.01	(0.84, 1.20)	1.01	(0.84, 1.20)	1.01	(0.84, 1.20)
Zone Entropy	1.03	(0.92, 1.16)	1.10	(0.94, 1.28)	1.10	(0.94, 1.28)	1.10	(0.94, 1.28)
Gray Level Variance	1.18	(1.07, 1.31)	1.20	(1.06, 1.35)	1.20	(1.06, 1.35)	1.20	(1.06, 1.35)
Large Area High Gray	1.11	(1.01, 1.22)	1.12	(1.01, 1.25)	1.12	(1.01, 1.25)	1.12	(1.01, 1.25)
Level Emphasis								

Note: HR, Hazard Ratio; CI, 95% Confidence Interval; DiD, Disorders.

## Appendix A. Demographic and Clinical Predictors

### A.1. Neighborhood Socioeconomic Status.

We defined four composite measures of neighborhood socioeconomic status at the US census tract-level based on patient residences (Salerno, Zhao, et al., 2021). These composites, derived from the National Neighborhood Data Archive, measured a neighborhood’s (1) affluence, (2) disadvantage, (3) ethnic immigrant concentration, and (4) education, and were defined in the average proportion of adults within a census tract fall meeting each respective measure’s criteria. Each measure was aggregated and was further categorized by quartiles (Table A2).

**Affluence:** the proportion of households with income greater than $75K, proportion of the population aged 16+ employed in professional or managerial occupations, and proportion of adults with bachelor’s degrees or higher.
**Disadvantage:** the proportion of non-Hispanic Black, proportion of female-headed families with children, proportion of households with public assistance income or food stamps, proportion of families with income below the federal poverty level, and proportion of the population aged 16+ unemployed.
**Ethnic Immigrant Concentration:** the proportion of Hispanic and proportion of foreign born.
**Education:** the proportion of adults with less than a high school diploma.

### A.2. Elixhauser Comorbidity Conditions.

Table A1 lists the comorbidity conditions considered as risk factors in this analysis and the corresponding ICD-10 codes used to define them. Each comorbidity was coded as a binary indicator, flagging whether a patient carried any ICD-10 code associated with the condition at baseline.

**Table A1.** Elixhauser comorbidity conditions and associated ICD-10 codes.

Comorbidity Condition	ICD-10 Codes
Congestive Heart Failure	I09.9, I11.0, I13.0, I13.2, I25.5, I42.0, I42.5-I42.9, I43.x, I50.x, P29.0
Cardiac Arrhythmias	I44.1-I44.3, I45.6, I45.9, I47.x-I49.x, R00.0, R00.1, R00.8, T82.1, Z45.0, Z95.0
Valvular Disease	A52.0, I05.x-I08.x, I09.1, I09.8, I34.x-I39.x, Q23.0-Q23.3, Z95.2-Z95.4
Pulmonary Circulation Disorders	I26.x, I27.x, I28.0, I28.8, I28.9
Peripheral Vascular Disorders	I70.x, I71.x, I73.1, I73.8, I73.9, I77.1, I79.0, I79.2, K55.1, K55.8, K55.9, Z95.8, Z95.9
Hypertension, Uncomplicated	I10.x
Hypertension, Complicated	I11.x-I13.x, I15.x
Paralysis	G04.1, G11.4, G80.1, G80.2, G81.x, G82.x, G83.0-G83.4, G83.9
Neurological Disorders	G10.x-G13.x, G20.x-G22.x, G25.4, G25.5, G31.2, G31.8, G31.9, G32.x, G35.x-G37.x, G40.x, G41.x, G93.1, G93.4, R47.0, R56.x
Chronic Pulmonary Disease	I27.8, I27.9, J40.x-J47.x, J60.x-J67.x, J68.4, J70.1, J70.3
Diabetes, Uncomplicated	E10.0, E10.1, E10.9, E11.0, E11.1, E11.9, E12.0, E12.1, E12.9, E13.0, E13.1, E13.9, E14.0, E14.1, E14.9
Diabetes, Complicated	E10.2-E10.8, E11.2-E11.8, E12.2-E12.8, E13.2-E13.8, E14.2-E14.8
Hypothyroidism	E00.x-E03.x, E89.0
Renal Failure	I12.0, I13.1, N18.x, N19.x, N25.0, Z49.0-Z49.2, Z94.0, Z99.2
Liver Disease	B18.x, I85.x, I86.4, I98.2, K70.x, K71.1, K71.3-K71.5, K71.7, K72.x-K74.x, K76.0, K76.2-K76.9, Z94.4 Peptic ulcer disease, excluding bleeding: K25.7, K25.9, K26.7, K26.9, K27.7, K27.9, K28.7, K28.9
Lymphoma	C81.x-C85.x, C88.x, C96.x, C90.0, C90.2
Metastatic Cancer	C77.x-C80.x
Solid Tumour without Metastasis	C00.x-C26.x, C30.x-C34.x, C37.x-C41.x, C43.x, C45.x-C58.x, C60.x-C76.x, C97.x
Rheumatoid Arthritis/Collagen Vascular	L94.0, L94.1, L94.3, M05.x, M06.x, M08.x, M12.0, M12.3, M30.x,
Diseases	M31.0-M31.3, M32.x-M35.x, M45.x, M46.1, M46.8, M46.9
Coagulopathy	D65-D68.x, D69.1, D69.3-D69.6
Obesity	E66.x
Weight Loss	E40.x-E46.x, R63.4, R64
Fluid and Electrolyte Disorders	E22.2, E86.x, E87.x
Blood Loss Anaemia	D50.0
Deficiency Anaemia	D50.8, D50.9, D51.x-D53.x
Alcohol Abuse	F10, E52, G62.1, I42.6, K29.2, K70.0, K70.3, K70.9, T51.x, Z50.2, Z71.4, Z72.1
Drug Abuse	F11.x-F16.x, F18.x, F19.x, Z71.5, Z72.2
Psychoses	F20.x, F22.x-F25.x, F28.x, F29.x, F30.2, F31.2, F31.5
Depression	F20.4, F31.3-F31.5, F32.x, F33.x, F34.1, F41.2, F43.2

**TABLE A2.** Summary of descriptive characteristics for the study population of 2,289 patients and stratified by predominant COVID-19 variant at diagnosis.

Characteristic	Overall^1^ n = 2,289	Original^1^ n = 1,528	Alpha^1^ n = 320	Delta^1^ n = 441	*p*-value^2^
Age, years	61 (43, 72)	62 (47, 73)	59 (36, 70)	59 (38, 72)	<0.001
Sex					0.8
Female	938 (41%)	630 (41%)	126 (39%)	182 (41%)	
Male	1,351 (59%)	898 (59%)	194 (61%)	259 (59%)	
Race					<0.001
White	1,610 (70%)	1,030 (67%)	229 (72%)	351 (80%)	
Black	436 (19%)	326 (21%)	58 (18%)	52 (12%)	
Other/Unknown	243 (11%)	172 (11%)	33 (10%)	38 (8.6%)	
Ethnicity					0.9
Hispanic or Latino	101 (4.4%)	64 (4.2%)	15 (4.7%)	22 (5.0%)	
Non-Hispanic or Latino	2,104 (92%)	1,409 (92%)	291 (91%)	404 (92%)	
Refused/Unknown	84 (3.7%)	55 (3.6%)	14 (4.4%)	15 (3.4%)	
Body Mass Index	29 (24, 34)	29 (25, 34)	29 (23, 33)	28 (24, 34)	0.3
Alcohol Abuse	310 (14%)	222 (15%)	36 (11%)	52 (12%)	0.15
Blood Loss, Anemia	519 (23%)	386 (25%)	66 (21%)	67 (15%)	<0.001
Cardiac Arrhythmias	1,795 (78%)	1,230 (80%)	238 (74%)	327 (74%)	0.003
Chronic Pulmonary Disease	1,094 (48%)	790 (52%)	133 (42%)	171 (39%)	<0.001
Coagulopathy	944 (41%)	672 (44%)	121 (38%)	151 (34%)	<0.001
Congestive Heart Failure	1,029 (45%)	733 (48%)	135 (42%)	161 (37%)	<0.001
Deficiency, Anemia	725 (32%)	551 (36%)	80 (25%)	94 (21%)	<0.001
Depression	1,001 (44%)	717 (47%)	139 (43%)	145 (33%)	<0.001
Diabetes	1,048 (46%)	755 (49%)	126 (39%)	167 (38%)	<0.001
Drug Abuse	416 (18%)	299 (20%)	49 (15%)	68 (15%)	0.050
Fluid and Electrolyte Disorders	1,768 (77%)	1,218 (80%)	217 (68%)	333 (76%)	<0.001
Hypertension	1,712 (75%)	1,207 (79%)	226 (71%)	279 (63%)	<0.001
Hypothyroidism	528 (23%)	379 (25%)	70 (22%)	79 (18%)	0.009
Liver Disease	708 (31%)	506 (33%)	88 (28%)	114 (26%)	0.005
Lymphoma	225 (9.8%)	156 (10%)	37 (12%)	32 (7.3%)	0.10
Metastatic Cancer	516 (23%)	355 (23%)	78 (24%)	83 (19%)	0.10
Obesity	1,137 (50%)	783 (51%)	163 (51%)	191 (43%)	0.012
Neurological Disorders	802 (35%)	587 (38%)	98 (31%)	117 (27%)	<0.001
Paralysis	329 (14%)	243 (16%)	44 (14%)	42 (9.5%)	0.003
Peptic Ulcer Disease, Excluding Bleeding	290 (13%)	205 (13%)	45 (14%)	40 (9.1%)	0.039
Peripheral Vascular Disorders	917 (40%)	653 (43%)	129 (40%)	135 (31%)	<0.001
Psychoses	294 (13%)	221 (14%)	36 (11%)	37 (8.4%)	0.002
Pulmonary Circulation Disorders	778 (34%)	555 (36%)	104 (32%)	119 (27%)	0.001
Renal Failure	1,017 (44%)	757 (50%)	121 (38%)	139 (32%)	<0.001
Autoimmune Diseases	407 (18%)	293 (19%)	56 (18%)	58 (13%)	0.014
Solid Tumor Without Metastasis	520 (23%)	360 (24%)	73 (23%)	87 (20%)	0.2
Valvular Disease	698 (30%)	505 (33%)	85 (27%)	108 (24%)	<0.001
Weight Loss	822 (36%)	614 (40%)	100 (31%)	108 (24%)	<0.001
Oxygen Saturation	95.57 (93.75, 97.28)	95.56 (93.80, 97.29)	95.53 (93.96, 97.50)	95.67 (93.50, 97.10)	0.7
Temperature	98.30 (97.93, 98.81)	98.33 (97.95, 98.93)	98.18 (97.87, 98.59)	98.30 (97.92, 98.69)	<0.001
Respiratory Rate	19.7 (17.8, 24.0)	19.5 (17.8, 23.7)	19.6 (17.3, 23.2)	20.5 (18.0, 24.9)	0.003
Diastolic Blood Pressure	67 (61, 74)	67 (61, 74)	67 (60, 74)	66 (60, 74)	0.4
Systolic Blood Pressure	122 (110, 137)	123 (110, 138)	121 (110, 136)	120 (109, 135)	0.3
Heart Rate	85 (73, 98)	85 (74, 98)	84 (73, 95)	85 (73, 99)	0.5
Respiratory Support					0.6
No Respiratory Support	1,376 (71%)	918 (72%)	189 (69%)	269 (70%)	
Respiratory Support	563 (29%)	362 (28%)	84 (31%)	117 (30%)	
Affluence Quartile					<0.001
1	530 (25%)	391 (27%)	66 (22%)	73 (18%)	
2	496 (23%)	327 (23%)	72 (24%)	97 (23%)	
3	547 (25%)	333 (23%)	79 (27%)	135 (32%)	
4	590 (27%)	398 (27%)	81 (27%)	111 (27%)	
Disadvantage Quartile					<0.001
1	659 (30%)	398 (27%)	103 (35%)	158 (38%)	
2	547 (25%)	354 (24%)	79 (27%)	114 (27%)	
3	445 (21%)	316 (22%)	49 (16%)	80 (19%)	
4	512 (24%)	381 (26%)	67 (22%)	64 (15%)	
Ethnic Immigration Quartile					0.011
1	1,004 (46%)	643 (44%)	137 (46%)	224 (54%)	
2	777 (36%)	533 (37%)	114 (38%)	130 (31%)	
3	336 (16%)	240 (17%)	38 (13%)	58 (14%)	
4	46 (2.1%)	33 (2.3%)	9 (3.0%)	4 (1.0%)	
Education Quartile					<0.001
1	750 (35%)	478 (33%)	113 (38%)	159 (38%)	
2	708 (33%)	450 (31%)	100 (34%)	158 (38%)	
3	518 (24%)	378 (26%)	59 (20%)	81 (19%)	
4	187 (8.6%)	143 (9.9%)	26 (8.7%)	18 (4.3%)	
Vaccination Status					0.002
Not Vaccinated	1,914 (84%)	1,255 (82%)	262 (82%)	397 (90%)	
Partially Vaccinated	84 (3.7%)	61 (4.0%)	15 (4.7%)	8 (1.8%)	
Fully Vaccinated	291 (13%)	212 (14%)	43 (13%)	36 (8.2%)	

1 Median (Q1, Q3); n (%)

2 Kruskal-Wallis rank sum test; Pearson’s Chi-squared test

## Appendix B. Simulations to Assess the Robustness of Mean Imputation

After data preprocessing, variables with no more than 30% missing data were included in our analysis. As shown in Table B1, diastolic blood pressure (78.90% missing), systolic blood pressure (78.90% missing), religion (36.30% missing), and marital status (32.90% missing) had more than 30% missing data. They were excluded in the data preprocessing procedure. Although the preferred language only had 0.57% missing data, it was also excluded, as 97% of non-missing cases were English. For computational convenience, missing values in the included variables were imputed using mean or mode, as described in Section 2.2. The missing percentages among these variables range from 4.19% (BMI) to 19.05% (temperature); see Table B1.

**Table B1.** Summary of missingness for the study population of 2,289 patients. Predictors with no missing values were not included in this table for conciseness. Predictors with greater than 30% missing data were excluded from the analysis. Missing values of predictors included in the analysis were imputed by mean (for continuous variables) or mode (for categorical variables).

Characteristic	Number (%) Missing	How Handled
Diastolic Blood Pressure (Invasive from Arterial Line)	1,806 (78.90%)	Excluded
Systolic Blood Pressure (Invasive from Arterial Line)	1,806 (78.90%)	Excluded
Religion	831 (36.30%)	Excluded
Marital Status	753 (32.90%)	Excluded
Temperature	436 (19.05%)	Imputed
Oxygen Saturation	402 (17.56%)	Imputed
Respiratory Rate	400 (17.47%)	Imputed
Heart Rate	393 (17.17%)	Imputed
Respiratory Support	350 (15.29%)	Imputed
Diastolic Blood Pressure (Non-Invasive from Cuff)	162 ( 7.08%)	Imputed
Systolic Blood Pressure (Non-Invasive from Cuff)	162 ( 7.08%)	Imputed
Affluence Quartile	126 ( 5.50%)	Imputed
Disadvantage Quartile	126 ( 5.50%)	Imputed
Ethnic Immigration Quartile	126 ( 5.50%)	Imputed
Education Quartile	126 ( 5.50%)	Imputed
Body Mass Index	96 ( 4.19%)	Imputed
Preferred Language^1^	13 ( 0.57%)	Excluded

1 Preferred language was additionally excluded as a predictor, as 97% of the non-missing cases were English speaking.

We assessed the performance of the mean imputation by varying the percentage of missingness for each covariate from the ideal case (0%) to 10% and 20%, corresponding respectively to the average and maximal missingness percentage among the included variables in the real data analysis. First, we generated the data as done in Section 5. Then we additionally simulated missingness under the assumption of missing at random (MAR), i.e., the missing patterns solely depend on the observed data (Rubin, 1976). Let $R_{i,j}$ be the missing indicator for covariate j of subject $i,X_{i,j}$, e.g., $R_{i,j}=1$ if $X_{i,j}$ is observed, and = 0 otherwise. For $j=1,\ldots ,22,R_{i,j}$ is generated from the following model:

$$\text{logit}\left\{\text{Pr}\left(R_{i,j}=1|X_{i,\left(-j\right)}\right)\right\}=a_{0}+X_{i,1}\gamma_{1}+\cdots +X_{i,j-1}\gamma_{j-1}+X_{i,j+1}\gamma_{j+1}+\cdots +X_{i,22}\gamma_{22},$$

where $logit(x)=ln\{x/(1-x)\},X_{i,(-j)}$ is $X_{i}$ excluding variable j, the γ coefficients were generated from 𝒰(−a,a), and a and $a_{0}$ were chosen to achieve approximate missingness rates of 10% or 20%, corresponding respectively to the average and maximal missingness percentage among the included variables in the real data analysis. Since the covariates were generated through a multivariate normal distribution with non-zero correlations, mean imputation may not be the most optimal method for imputation. However, due to its computational efficiency, we aimed to evaluate its performance, particularly in situations where the proportion of missing data is not excessively high. The results suggest that the conclusions derived from scenarios with complete data hold true even in the presence of missing data, even when imputed using means. For example, if the linear assumption holds, it is evident that all prediction models based on mean imputed values exhibit commendable performance, with the Cox model with linear log hazards consistently outperforming the others across all levels of censoring rates when the missing percentage is less than 10%. The performance of the proposed weighted ensemble method closely aligns with that of the Cox model. When the missing percentage is 20%, and the censoring rate is less than 80%, the performance of the weighted ensemble method is better than the other models. On the other hand, if the linear assumption fails, the weighted ensemble averaging method with mean imputed values consistently outperforms all other competing methods, achieving the highest median C-index across all levels of censoring rates. However, the effectiveness of predictive models based on mean imputation diminishes as the proportion of missing data increases. The decline is expected, given that the covariates were generated from a multivariate normal distribution with non-zero correlations. Mean imputation may overlook such information.

**Table B2.** Comparisons of median C-indices of six machine learning algorithms across varying censoring rates (40% to 80%) under a linear log hazard model with various proportions of missing data for each covariate (0%, 10%, 20%). The table displays median C-index values from 100 experiments, along with their interquartile ranges.

	Censoring Rate			
	40%	60%	70%	80%
**0% Missing**				
Cox Model with Linear Log Hazards	85.9 (1.6)	87.9 (1.6)	88.0 (1.8)	90.1 (1.5)
Survival Support Vector Machines	85.8 (1.6)	87.8 (1.6)	87.8 (1.9)	89.8 (1.4)
Survival Gradient Boosting	84.6 (1.7)	86.3 (1.8)	86.1 (2.1)	88.0 (1.9)
Random Survival Forests	83.1 (2.0)	84.8 (1.9)	85.3 (2.3)	86.9 (2.3)
Naive Ensemble Averaging	83.1 (2.0)	85.0 (1.9)	85.5 (2.3)	87.3 (2.1)
Weighted Ensemble Averaging	85.6 (1.4)	87.4 (1.7)	87.4 (1.8)	89.5 (1.5)
**10% Missing**				
Cox Model with Linear Log Hazards	83.6 (2.3)	85.2 (2.1)	85.8 (2.4)	87.7 (2.6)
Survival Support Vector Machines	83.6 (2.1)	85.2 (2.1)	85.7 (2.3)	87.6 (2.8)
Survival Gradient Boosting	82.2 (2.2)	83.9 (2.3)	84.2 (2.6)	85.9 (2.7)
Random Survival Forests	80.9 (2.1)	82.6 (2.5)	83.3 (2.9)	84.9 (2.7)
Naive Ensemble Averaging	81.0 (2.1)	82.8 (2.5)	83.4 (2.8)	85.2 (2.8)
Weighted Ensemble Averaging	83.3 (1.9)	85.1 (2.1)	85.4 (2.5)	87.2 (2.6)
**20% Missing**				
Cox Model with Linear Log Hazards	81.5 (2.2)	83.5 (3.4)	83.2 (3.3)	86.1 (3.8)
Survival Support Vector Machines	81.7 (2.2)	83.7 (2.6)	83.3 (3.2)	85.9 (3.3)
Survival Gradient Boosting	80.7 (2.3)	82.9 (3.0)	82.7 (3.0)	84.7 (3.2)
Random Survival Forests	79.5 (2.6)	81.5 (2.9)	81.7 (2.9)	83.2 (3.1)
Naive Ensemble Averaging	79.6 (2.6)	81.6 (2.8)	81.8 (2.9)	83.6 (3.0)
Weighted Ensemble Averaging	81.8 (2.4)	83.9 (2.7)	83.4 (2.4)	85.9 (2.8)

**Table B3.** Comparisons of median C-indices of six machine learning algorithms across varying censoring rates (40% to 80%) under a nonlinear log hazard model with various proportions of missing data for each covariate (0%, 10%, 20%). The table displays median C-index values from 100 experiments, along with their interquartile ranges.

	Censoring Rate			
	40%	60%	70%	80%
**0% Missing**				
Cox Model with Linear Log Hazards	65.6 (2.0)	67.8 (3.0)	69.0 (2.9)	72.5 (3.1)
Survival Support Vector Machines	73.0 (1.7)	74.6 (1.4)	76.3 (1.8)	78.7 (2.4)
Survival Gradient Boosting	72.6 (1.3)	74.2 (1.6)	75.7 (2.1)	78.0 (2.3)
Random Survival Forests	71.5 (1.5)	73.3 (1.6)	75.2 (2.0)	77.5 (1.9)
Naive Ensemble Averaging	71.5 (1.5)	73.4 (1.6)	75.3 (2.0)	77.6 (1.9)
Weighted Ensemble Averaging	73.1 (1.1)	74.8 (1.4)	76.6 (1.6)	78.9 (1.6)
**10% Missing**				
Cox Model with Linear Log Hazards	64.5 (1.8)	66.3 (3.3)	67.9 (3.5)	70.8 (2.8)
Survival Support Vector Machines	70.7 (1.9)	72.3 (2.2)	73.5 (1.7)	75.9 (3.0)
Survival Gradient Boosting	69.9 (2.1)	71.4 (2.0)	73.0 (2.8)	74.7 (2.7)
Random Survival Forests	69.6 (1.6)	71.3 (2.5)	72.8 (2.8)	75.1 (2.3)
Naive Ensemble Averaging	69.6 (1.6)	71.4 (2.5)	72.8 (2.9)	75.2 (2.4)
Weighted Ensemble Averaging	70.9 (1.4)	72.5 (2.1)	74.4 (2.4)	76.4 (2.5)
**20% Missing**				
Cox Model with Linear Log Hazards	63.8 (2.0)	65.6 (3.2)	66.0 (3.4)	69.4 (4.0)
Survival Support Vector Machines	68.9 (2.1)	70.6 (2.4)	71.5 (2.3)	73.5 (3.9)
Survival Gradient Boosting	67.8 (1.9)	70.1 (2.9)	70.0 (3.3)	72.9 (3.9)
Random Survival Forests	68.1 (2.0)	69.7 (2.0)	70.9 (3.3)	73.0 (3.9)
Naive Ensemble Averaging	68.1 (2.0)	69.8 (2.0)	71.0 (3.3)	73.1 (4.0)
Weighted Ensemble Averaging	69.2 (1.5)	71.0 (2.2)	72.0 (2.9)	74.5 (3.5)
